# Real Time RT-PCR Assays for Detection and Typing of African Horse Sickness Virus

**DOI:** 10.1371/journal.pone.0093758

**Published:** 2014-04-10

**Authors:** Katarzyna Bachanek-Bankowska, Sushila Maan, Javier Castillo-Olivares, Nicola M. Manning, Narender Singh Maan, Abraham C. Potgieter, Antonello Di Nardo, Geoff Sutton, Carrie Batten, Peter P. C. Mertens

**Affiliations:** 1 Vector-borne Viral Diseases Programme, The Pirbright Institute, Pirbright, Surrey, United Kingdom; 2 Deltamune (Pty) Ltd, Lyttelton, Centurion, South Africa; 3 Department of Biochemistry, Centre for Human Metabonomics, North-West University, Private Bag X6001, Potchefstroom, South Africa; 4 Division of Structural Biology, Wellcome Trust Centre for Human Genetics, University of Oxford, Oxford, United Kingdom; University of Brighton, United Kingdom

## Abstract

Although African horse sickness (AHS) can cause up to 95% mortality in horses, naïve animals can be protected by vaccination against the homologous AHSV serotype. Genome segment 2 (Seg-2) encodes outer capsid protein VP2, the most variable of the AHSV proteins. VP2 is also a primary target for AHSV specific neutralising antibodies, and consequently determines the identity of the nine AHSV serotypes. In contrast VP1 (the viral polymerase) and VP3 (the sub-core shell protein), encoded by Seg-1 and Seg-3 respectively, are highly conserved, representing virus species/orbivirus-serogroup-specific antigens. We report development and evaluation of real-time RT-PCR assays targeting AHSV Seg-1 or Seg-3, that can detect any AHSV type (virus species/serogroup-specific assays), as well as type-specific assays targeting Seg-2 of the nine AHSV serotypes. These assays were evaluated using isolates of different AHSV serotypes and other closely related orbiviruses, from the ‘Orbivirus Reference Collection’ (ORC) at The Pirbright Institute. The assays were shown to be AHSV virus-species-specific, or type-specific (as designed) and can be used for rapid, sensitive and reliable detection and identification (typing) of AHSV RNA in infected blood, tissue samples, homogenised *Culicoides*, or tissue culture supernatant. None of the assays amplified cDNAs from closely related heterologous orbiviruses, or from uninfected host animals or cell cultures.

## Introduction

African horse sickness (AHS) is a rapidly lethal disease of horses, caused by the African horse sickness virus (AHSV), which is transmitted primarily via the bite of infected *Culicoides* midges [1]. There are nine distinct serotypes of AHSV (AHSV-1 to AHSV-9) that can be distinguished in serum neutralisation tests by the specificity of their reactions with neutralising antibodies [2], [3]. These different serotypes are all classified within the species, *African horse sickness virus,* genus *Orbivirus*, family *Reoviridae* [4]. Clinical signs of AHS can be very severe in populations of naïve horses, where mortality rates can exceed 95%. However, zebras, donkeys and mules develop milder disease which can easily be missed [5]. Due to its economic importance and the occurrence of large ‘transboundary’ outbreaks AHS has been ‘listed’ by the World Organisation for Animal Health [6].

Like other orbiviruses, the AHSV genome consists of 10-segments of linear double-stranded (ds) RNA (genome segments 1 to 10 [Seg-1 to Seg-10]), most of which encode a single viral protein [4], [7], [8]. The outer-most protein of the AHSV capsid and the primary determinant of serotype is VP2, encoded by Seg-2. Consequently Seg-2 and VP2 of AHSV (like those of the *Orbivirus* type species, ‘*Bluetongue virus’* [BTV]) both vary in a serotype-specific manner and are the most variable AHSV genome segment and protein respectively [9]–[12].

In contrast, the viral polymerase VP1 (encoded by Seg-1), and the sub-core shell protein VP3 (encoded by Seg-3) are the two most conserved of the orbivirus structural-proteins, showing sequence variations that correlate with the virus species/serogroup and with the geographic origins of the virus isolate [13]–[15]. The ability of orbiviruses within a single virus species to exchange genome segments by reassortment has resulted in an absence of any detectable correlation between sequence variation in Seg-1 (VP1) or Seg-3 (VP3-T2) and the virus serotype.

At present, AHSV is considered to be endemic only in sub-Saharan Africa, with outbreaks involving all nine serotypes of AHSV occurring in South Africa [5], [16]. Until 2007, AHSV-9 was the main serotype circulating in the equine populations of Central Africa [1]. However, other AHSV serotypes have subsequently also been reported in the region, including AHSV-2 in Senegal and Nigeria in 2007, in Ethiopia in 2008 and Ghana in 2010 [17]–[20]; AHSV-4 in Kenya in 2007 [21], and AHSV-7 in Senegal in 2007 [21]. In 2010 an outbreak involving multiple serotypes of AHSV also took place in Ethiopia [22], [23].

However, AHSV has caused devastating outbreaks in indigenous horse populations outside of its current endemic zone, including AHSV-9 in the Middle East, India and Pakistan (1959–1961) and in North Africa and Europe (1965) [1]. Importation of an infected zebra from Namibia to Madrid in 1987, led to an outbreak caused by AHSV-4 in Spain, that spread to Portugal and North Africa (1987–1990) [24]–[26].

Since 1998, multiple different serotypes of bluetongue virus (BTV), which are transmitted by similar *Culicoides* vectors to AHSV, have emerged in Southern Europe [27], leading up to the very severe outbreak caused by BTV-8 which started in 2006, the first ever recorded in northern Europe [28]. The virus overwintered and spread over most of Europe causing massive losses to the livestock industries of several countries over a number of years [29], [30]. Phylogenetic analyses showed that the northern European strain of BTV-8 is most closely related to a strain from Central Africa [31], although the route of introduction has not yet been identified.

Along with the recent disease outbreak caused by Schmallenberg virus [32], [33], this demonstrates the capacity for *Culicoides* transmission and the risk posed by these arboviruses in the region. With on-going climate change and increasing global trade, the distribution of vector-borne diseases (including the orbiviruses) is also considered likely to change [27], [34], [35], suggesting that the AHSV endemic regions within Africa could expand, and the virus could again emerge to threaten horse populations in other parts of the world. Previous AHS outbreaks have spread very rapidly and could persist for extended periods (years) in previously non-endemic areas, causing extensive damage to equine industries by direct losses and restrictions on animal movements [24]–[26], [36].

Live-attenuated AHSV vaccines are currently available from Onderstepoort Biological Products (OBP), South Africa [5]. These strains are currently used in AHSV endemic areas (in Africa), as part of polyvalent vaccine preparations. However, their use risks introduction of additional AHSV serotypes into the vaccinated region, with potential for reassortment with other vaccine and/or wild-type ‘field’ strains. Alternative vaccine candidates are in the process of being developed, which include individual AHSV proteins, delivered by recombinant poxviruses [37]–[40].

There is a need for accurate, reliable and rapid diagnostic systems for AHS, for use in surveillance programmes and identification of specific AHSV serotypes in clinical samples, to support the design and rapid deployment of appropriate control/vaccination strategies. These diagnostic systems are also important to demonstrate absence of the virus in individual animals or animal products (for safe movements/export/import), as well as for declaration of a ‘virus free’ status, especially after outbreaks in non-endemic countries or zones (OIE, 2011).

Historically, members of the AHSV serogroup/virus-species have been identified by serological methods, including complement fixation (CF) and ELISA tests [41]–[43]. AHSV virus-species-specific antibodies can also be detected by cELISA [44], [45]. However, these methods are labour intensive and require virus isolation and/or access to standard reagents (antibodies and antigens) that may themselves represent a potential biosecurity risk.

AHSV RNA can be detected by amplification in conventional or real-time RT-PCR assays. Different ‘conserved’ AHSV genome segments have previously been targeted by conventional RT-PCR assays, including the genes encoding VP3, VP7, NS1 and NS2 [46]–[50]. Post amplification analyses of conventional PCR assays involve agarose gels electrophoresis (AGE). This could lead to contamination and potentially false positive reactions in subsequent assays using the same laboratory space. In contrast, the design of real-time RT-PCR assays allows detection of the amplified target in a closed tube format, which significantly improves sample throughput and reduces contamination risk (false positives). These assays also offer enhanced sensitivity over the conventional PCR methods. Several real-time RT-PCR assays for detection of different AHSV genome segments have already been described, including those encoding VP7, VP7 & NS2, NS1 and NS2 genes [51]–[56].

The “gold standard” method for the determination of AHSV serotype is the virus neutralisation test (VNT) where the specificity of reactions between the virus and a panel of reference antisera, representing each of the known serotypes, is tested in tissue cultures [2], [3]. However, these serotyping assays are labour intensive, time consuming, require prior virus isolation and can sometimes give inconclusive results. They are also dependent on availability of reference virus strains (as controls) and reference antisera which are highly characterised and may therefore be difficult to obtain. These assays may also require disease-secure laboratory facilities for safe handling the live virus.

Molecular methods for determination of AHSV type, have recently been developed based on detection of Seg-2, including both conventional RT-PCR [57], [58] and probe-hybridisation methods [59], [60].

This paper describes the development and evaluation of real-time RT-PCR assays for detection and typing of AHSV. Two independent virus-species-specific assays, each of which detects all nine serotypes of AHSV, were designed to amplify specific regions of Seg-1 or Seg-3 respectively. A set of nine individual ‘typing’ assays were also designed to detect and identify Seg-2 of each AHSV serotype. Assay specificity was evaluated using a wide range of AHSV isolates from the Orbivirus Reference Collection (ORC), including reference, vaccine and field strains [61].

## Materials and Methods

### Primers and Probe Design

The design of primers and probes for the detection and typing of AHSV was based on Seg-1, Seg-2 and Seg-3 nucleotide sequences of the AHSV reference strains, that were generated during this study (as previously described [62]) and nucleotide sequences available in the public domain (GenBank) (Table 1 and Figure S1 in File S1).

**Table 1:** Sequence data used to design real time RT-PCR assays.

Virus serotype	Origin	ORC reference number^2^	Seg-1 Acc. number	Seg-2 Acc. number	Seg-3 Acc. number
**Reference strains** 1					
AHSV-1	Republic of South Africa	RSArrah/01*	KF446265	KF446274	KF446256
AHSV-2	Republic of South Africa	RSArrah/02*	KF446266	KF446275	KF446257
AHSV-3	Republic of South Africa	RSArrah/03*	KF446267	KF446276	KF446257
AHSV-4	Spain	SPArrah/04*	KF446268	KF446277	KF446259
AHSV-5	Republic of South Africa	RSArrah/05*	KF446269	KF446278	KF446260
AHSV-6	Republic of South Africa	RSArrah/06*	KF446270	KF446279	KF446261
AHSV-7	Kenya	KENrrah/07*	KF446271	KF446280	KF446262
AHSV-8	Republic of South Africa	RSArrah/08*	KF446272	KF446281	KF446263
AHSV-9	Pakistan	PAKrrah/09*	KF446273	KF446282	KF446264
**Sequence data from international databases**					
AHSV-1			NC006021 FJ011107 FJ183364 AM883164	AY163329 FJ011108 AM883165	AM883166 FJ011109 EU303138
AHSV-2			FJ196584	AY163332 FJ196585	EU303139
AHSV-3				DQ868772 U01832 Z26316	EU303136 EU303135 EU303134 EU303133 EU303132 EU303140
AHSV-4				EU046574 DQ868773 U21956	EU303141 EU303137 D26572
AHSV-5				AY163331	
AHSV-6				DQ868774 AF021235	EU303142 AF021236
AHSV-7				DQ118706 DQ118705 DQ118704 DQ118703 AY159954 AY159953 AY159952 AY159951 AY159950 AY159949 AY159948 AY159947 AY159946 AY159945 AY159944 AY159943 AY159942 AY159941 AY159940 AY163330	
AHSV-8				DQ868775 AY163333	EU303143
AHSV-9			U94887	DQ868776 AF043926	

*Isolates sequenced as part of this study.

1 Set of nine monotypic AHSV reference strains (Bachanek-Bankowska et al – in preparation).

2 Orbivirus reference collection (ORC), The Pirbright Institute.

Sequences for genome segments 1, 2 and 3, were aligned and analysed using MEGA v. 4 [63]. Conserved regions in Seg-1 and Seg-3 respectively were identified across all nine serotypes allowing primers and probes to be designed for separate assays, in accordance with TaqMan specifications (Table 2 and Figure S1 in File S1). Genome segment 2 sequences were analysed collectively and separately for each of the nine AHSV serotypes. Unique regions in Seg-2 of each serotype were identified as targets for primers and probes (Table 2 and Figure S1 in File S1). The probes were labelled at their 3′ and 5′ ends with BHQ-1 (Black Hole Quencher-1) and FAM respectively. All oligonucleotides were synthesised by Eurogentec, UK.

**Table 2:** List of primers and probes for AHSV virus-species- and type-specific assays.

Assay Type	Oligo Name	Oligo Sequence (5′-3′)
Seg-1 based/virus-species-specific		
	AHSV/Seg-1/FP/1310-1329	CCTGAAGATATGTATACATC
	AHSV/Seg-1/RP/1444-1461	ACTAACGCTTTAATCCGC
	AHSV/Seg-1/P/1400-1423	CCCAACGCRAARGGTGGACGTGG
Seg-3 based/virus-species-specific		
	AHSV/Seg-3/FP/2030-2050	AGATATYGTAAGGTGGAGTCA
	AHSV/Seg-3/RP/2115-2136	CTAACATCAARTCTTCAAARTC
	AHSV/Seg-3/P/2085-2107	ATCGCCCAAGCTTCCCTATTCAA^#^
Seg-2 based/type-specific		
	AHSV/SRT-1/FP/1143-1163	GAATGGTAAGCTTTGGATTGA
	AHSV/SRT-1/RP/1225-1207	TTAGAGGCGCTCGGTTCTC
	AHSV/SRT-1/P/1165-1187	CATAAACAAACGGTGAGTGAGCA
	AHSV/SRT-2/FP/1763-1783	AGTGGACTTCGATTATAGATG
	AHSV/SRT-2/RP/1897-1878	CTGTCTGAGCGTTAACCTTC
	AHSV/SRT-2/P/1863-1842	TTCAACCGTCTCTCCGCCTCTC^#^
	AHSV/SRT-3/FP/809-829	TAGAAAGAATGATGAGCAGTG
	AHSV/SRT-3/RP/901-921	TAATGGAATGTCGCCTGTCTT
	AHSV/SRT-3/P/845-870	CCGTTATTGAGAGCGTCATAAGATTC
	AHSV/SRT-4/FP/1934-1956	GTTTCGTATCATATTGGTATAGA
	AHSV/SRT-4/RP/2079-2101	GATATATATATGTGGATGCATGG
	AHSV/SRT-4/P/1996-2020	CYGGAYATGAATGAGAAACAGAAGC
	AHSV/SRT-5/FP/1875-1895	GAGACACATCAAGGTTAAAGG
	AHSV/SRT-5/RP/2035-2014	CAGGATCAAACTGTGTATACTT
	AHSV/SRT-5/P/1964-1985	TTGAAGCAAGGAATCTATTGACTTT
	AHSV/SRT-6/FP/1285-1304	GTTGGTCATTGGGTCGATTG
	AHSV/SRT-6/RP/1505-1485	GACGAATTTGATCCCTTCTTG
	AHSV/SRT-6/P/1385-1408	TGATGTCGGGTATGAACAAACTGG
	AHSV/SRT-7/FP/1159-1179	TGGATCGAGCATAAGAAGAAG
	AHSV/SRT-7/RP/1282-1302	CCAATCAACCCARTGTGTAAC
	AHSV/SRT-7/P/1213-1237	ACCAAAATCGTCCGATGCTAGTGC
	AHSV/SRT-8/FP/2122-2141	GGTGAGCGGATTGTTGATAG
	AHSV/SRT-8T/RP/2293-2274	CTGAATCCACTCTATACCTC
	AHSV/SRT-8/P/2169-2146	CGACAAATCATACCACACGATCTC^#^
	AHSV/SRT-9/FP/1931-1952	TATCATATTGGTATCGAGTTCG
	AHSV/SRT-9/RP/2074-2054	AAGTTGATGCGTGAATACCGA
	AHSV/SRT-9/P/1978-2000	ACATCCTCAATCGAYCTCCTCTC^#^

The names of the oligonucleotide primers and probes indicate the group- or type-specificity of the assay. Seg-1 and Seg-3 indicate the group-specific assays while SRT-1 to SRT-9 indicate respective type-specific assays. FP, RP or P stand for ‘forward primer’, ‘reverse primer’ and ‘probe’ respectively. The numbers represent annealing positions on the genome segment. Probes indicated (^#^) are complementary to the negative strand.

The sequences of the different primers and probes were evaluated *in silico* to ensure no cross-reactions with closely related orbiviruses. Sequences for the relevant genome segments of all 22 orbivirus species of were used for *in silico* analysis [12], [13], [15], [64].

### Virus Isolates

A panel of 42 isolates representing all nine of the known AHSV serotypes, including monotypic reference, field and vaccine strains from different geographical locations, were used for initial validation of real-time RT-PCR assays (Table 3). Representative isolates of other orbiviruses were used to assess specificity of the virus-species-specific assays. All isolates used in the study were obtained from the ORC at The Pirbright Institute [61] and were grown in BHK-21 clone 13 cells (European Collection of Animal cell Cultures [ECACC–84100501]), Vero cell monolayers (ECACC–84113001), or in KC cells derived from *Culicoides sonorensis* [65]. Infected mammalian cells were harvested when 40–100% cytopathic effect (CPE) was observed, while KC cells were harvested 7 days post infection.

**Table 3:** AHSV isolates used to validate real time RT-PCR assays for AHSV.

Virus serotype	Origin	ORC reference number^1^	
**Reference strains** 2			
AHSV-1	Republic of South Africa	RSArah1/03*	
AHSV-2	Republic of South Africa	RSArah2/03*	
AHSV-3	Republic of South Africa	RSArah3/03*	
AHSV-4	Spain	SPArah4/03*	
AHSV-5	Republic of South Africa	RSArah5/03*	
AHSV-6	Republic of South Africa	RSArah6/03*	
AHSV-7	Kenya	KENrah7/03*	
AHSV-8	Republic of South Africa	RSArah8/03*	
AHSV-9	Pakistan	PAKrah9/03*	
**Field strains**			
AHSV-2	Ethiopia	ETH2010/01*	
AHSV-2	Ethiopia	ETH2010/20*	
AHSV-2	Ghana	GHA2010/01*	
AHSV-2	Ghana	GHA2010/02*	
AHSV-2	Ghana	GHA2010/03	
AHSV-2	Ghana	GHA2010/04	
AHSV-2	Ghana	GHA2010/05	
AHSV-2	Senegal	SEN2007/01	
AHSV-2	Senegal	SEN2007/02*	
AHSV-2	Senegal	SEN2007/03	
AHSV-2	Senegal	SEN2007/04*	
AHSV-2	Senegal	SEN2007/05*	
AHSV-4	Ethiopia	ETH2010/03	
AHSV-4	Ethiopia	ETH2010/04	
AHSV-4	Ethiopia	ETH2010/05*	
AHSV-4	Ethiopia	ETH2010/06	
AHSV-4	Ethiopia	ETH2010/07*	
AHSV-4	Kenya	KEN2007/01*	
AHSV-4	Spain	SPA1987/01*	
AHSV-6	Ethiopia	ETH2010/19*	
AHSV-7	Senegal	SEN2007/06*	
AHSV-8	Ethiopia	ETH2010/10*	
AHSV-8	Ethiopia	ETH2010/11*	
AHSV-9	Ethiopia	ETH2010/14	
AHSV-9	Ethiopia	ETH2010/15	
AHSV-9	Ethiopia	ETH2010/16*	
AHSV-9	Gambia	GAM2009/01*	
AHSV-9	Gambia	GAM2009/02*	
AHSV-9	Kenya	KEN2006/01*	
**Vaccine strains**			
AHSV-2+3	Ethiopia	ETH2010/02*	
AHSV-5+7	Ethiopia	ETH2010/09*	
AHSV-5+9	Ethiopia	ETH2010/18*	
AHSV-9	Senegal	SENvvv1/09*	

1 Orbivirus reference collection (ORC), The Pirbright Institute.

2 Set of nine monotypic AHSV reference strains (Bachanek-Bankowska et al – in preparation).

*Seg-2 of these isolates were only partially sequenced and compared to the reference strains of each serotype, to confirm RT-PCR results (data not shown).

The original diagnostic samples collected from horses, showing clinical signs of disease, were not for taken for research purposes but were collected as part of routine veterinary investigations carried out by qualified veterinarians in the respective countries of origin. The viruses studied here include isolates that were subsequently recovered from these diagnostic samples (from naturally infected animals - blood and/or spleen or lung), as well as the previously established reference strains of AHSV [66], [67]. No animals were infected, and no samples were taken from animals specifically for these studies. Further ethical approval was not therefore sought or obtained.

### RNA Sample Preparation

dsRNA was extracted either from tissue culture supernatant by BioRobot Universal (Qiagen, UK), or from AHSV infected cells (using Trizol Reagent, Invitrogen, UK) [68]. In the first method, 240 μl of infected-tissue-culture supernatant was added to 40 μl of protease (Qiagen, UK) and 360 μl of Lysis Buffer (Roche, UK). Total nucleic acid (50 μl) was extracted from this solution using the BioRobot platform UNIrcV23A V3.0 protocol. Each of the isolates was handled with care to avoid any cross-contamination. Total nucleic acid from uninfected tissue culture supernatants, horse blood or homogenised *Culicoides* was also extracted using BioRobot Universal (Qiagen, UK).

### RT-PCR

The different primers-probe sets were used to amplify a fragment of the targeted genome segment, using SuperScript III/Platinum Taq One-Step qRT-PCR Kit (Invitrogen, UK). In order to achieve low cycle threshold (Ct) values and high fluorescence signals, the individual steps of the method were optimised for each of the assays, including: primers concentrations, primers-probe ratios, magnesium concentration, reverse transcription temperature, reverse transcription period, annealing temperature and annealing period. The composition of the individual assays, after optimisation, is presented in Table 4. dsRNA samples were heat denaturated prior to addition to the reaction mix [69]. To avoid accidental contamination, assembly of the reaction mix was conducted in a ‘clean work’ dedicated laminar flow hood, separate to an RT-PCR assembly hood. Amplification for all of the assays was carried out in MX3005p (Stratagene, UK) using the same conditions: 55°C for 30 min, 95°C for 10 min followed by 45 cycles of 95°C for 30 s and 55°C for 1 min. Fluorescence was measured during the 55°C annealing/extension step. Cycle threshold (Ct) values were measured at the point at which the sample fluorescence signal crossed a threshold value (the background level). Negative results (for assays that did not exceed this level of signal) are reported as ‘No Ct’.

**Table 4:** RT-PCR assay composition.

Reagent	Assay detecting segment		
	Seg-1	Seg-3	Seg-2
Forward primer (10 μM)(μl)	2	1	2
Reverse primer (10 μM)(μl)	2	1	2
Probe (5 μM)(μl)	1.5	1	0.5
MgSO4*(μl)	0.5	1.5	1
SuperScript III RT/Platinum *Taq* Mix*(μl)	0.5	0.5	0.5
2x Reaction Mix*(μl)	12.5	12.5	10
Nuclease free water (μl)		1.5	
dsRNA(μl)	6	6	4
**total volume(μl)**	**25**	**25**	**20**

*SuperScript III/Platinum Taq One-Step qRT-PCR Kit (Invitrogen, UK).

### Diagnostic Sensitivity and Specificity

A panel of AHSV isolates from different geographical locations, including all nine serotypes (Table 3), was tested in duplicate, using both of the virus-species-specific assays targeting Seg-1 or Seg-3 respectively. The Seg-2 based assays (type-specific assays) were also tested with both homologous and heterologous serotypes to ensure specificity and diagnostic sensitivity. Representative isolates of other *Orbivirus* species, including BTV, *Epizootic haemorrhagic disease virus* (EHDV), *Equine encephalosis virus* (EEV) and *Peruvian horse sickness virus* (PHSV) were used to assess diagnostic specificity of Seg-1 and Seg-3 based assays, as listed in Table 5. To ensure that there were no false positives caused by cross-reactions with the host-species, total nucleic acid extracted from uninfected BHK and KC cell culture supernatants, horse blood (n = 8) and three individually homogenised *C. sonorensis*, were also tested with uniformly negative results (Table 5).

**Table 5:** Specificity of AHSV virus-species-specific assays (Seg-1 and Seg-3).

Virus species-serotype	Origin	ORC reference number	Ct values	
			Seg-1	Seg-3
Reference strain AHSV-1	RSA	RSArah1/03	17.36	19.13
Reference strain AHSV-2	RSA	RSArah2/03	16.56	16.02
Reference strain AHSV-3	RSA	RSArah3/03	21.1	22.02
Reference strain AHSV-4	RSA	SPArah4/03	14.37	16.09
Reference strain AHSV-5	RSA	RSArah5/03	19.02	20.52
Reference strain AHSV-6	RSA	RSArah6/03	20.00	19.81
Reference strain AHSV-7	Kenya	KENrah7/03	17.22	16.17
Reference strain AHSV-8	RSA	RSArah8/03	17.08	16.41
Reference strain AHSV-9	Pakistan	PAKrah9/03	16.73	12.63
EEV-1	RSA	RSA1967/03	No Ct	No Ct
EEV-2	RSA	RSA1971/06	No Ct	No Ct
EEV-3	RSA	RSA1974/06	No Ct	No Ct
EEV-4	RSA	RSA1976/03	No Ct	No Ct
EEV-5	RSA	RSA1976/06	No Ct	No Ct
EEV-6	RSA	RSA1991/03	No Ct	No Ct
PHSV	Peru	PER1997/01	No Ct	No Ct
EHDV-1	USA	USA2001/03	No Ct	No Ct
EHDV-5	Australia	AUS1977/01	No Ct	No Ct
EHDV-7	Australia	AUS1981/06	No Ct	No Ct
EHDV-7	Israel	ISR2006/01	No Ct	No Ct
BTV-16	Pakistan	RSArrrr/16	No Ct	No Ct
BTV-2	India	IND1982/01	No Ct	No Ct
BTV-9	Turkey	TUR1998/04	No Ct	No Ct
BTV-8	UK	UKG2007/64	No Ct	No Ct
BTV-1	RSA	RSArrrr/01	No Ct	No Ct
BTV-12	RSA	RSArrrr/12	No Ct	No Ct
*C. sonorensis*			No Ct	No Ct
Uninfected BHK cells			No Ct	No Ct
Uninfected Vero cells			No Ct	No Ct
Uninfected KC cells			No Ct	No Ct
Uninfected horse blood			No Ct	No Ct

Representatives of different serotypes and topotypes from five different *Orbivirus* species (AHSV, EEV, PHSV, EHDV and BTV) were tested to confirm the specificity of assays for AHSV dsRNA. Further details on these isolates can be obtained from ORC http://www.reoviridae.org/dsRNA_virus_proteins/ReoID/viruses-at-iah.htm [61]. EEV  =  Equine encephalosis virus; PHSV  =  Peruvian horse sickness virus; EHDV  =  Epizootic haemorrhagic disease virus; BTV  =  Bluetongue virus. RSA  =  Republic of South Africa.

### Assay Sensitivity and Efficiency

The analytical sensitivity of the virus-species-specific assays (Seg-1 and Seg-3) was assessed separately using two independent methods, either with a dilution series of the nine AHSV viruses, or with a dilution series of the quantified dsRNA genome of AHSV-1 (RSArah01/03). In the type-specific assays (Seg-2) these qualities were assessed by analysis of a dilution series of the homologous AHSV reference strain.

A tenfold dilution series of the nine monotypic (plaque purified) AHSV reference strains were prepared in cell culture medium, starting at a titre of 10^5^ TCID_50_/ml, down to 1 TCID_50_/ml (titrated in BHK cells). The viral dsRNA was extracted from the dilution series using BioRobot Universal (Qiagen, UK), as described above and tested in duplicate with each of the optimised assays.

To determine the analytical sensitivity of both virus-species-specific assays (Seg-1 and Seg-3) with quantified RNA, dsRNA standards were prepared as follows: viral dsRNA (RSArah01/03) was extracted as previously described [68], assessed for any ssRNA remains in 1% agarose gel electrophoresis and the concentration of dsRNA was determined with NanoDrop (Thermo Fisher Scientific, USA). The number of dsRNA copies was calculated with the formula: Y = (X/a×680)×6.022×10^23^, where: Y  =  molecules/μl; X = g/μl dsRNA; a  =  viral genome length in nucleotides; 680 is the average molecular weight per nucleotide of dsRNA.

To test the analytical sensitivity of each of the virus-species-specific assays, a 10-fold dilution series of dsRNA (10^9^ to 10^0^ copies per μl) was made in a sample of RNA extracted from uninfected BHK cells supernatant, then tested in quadruplicate.

In all the assays tested (Seg-1, Seg-3 and Seg-2), virus dilution series were used to generate standard curves by linear regression method setting the Ct values as dependent and the log-titre of virus dilution (TCID_50_/ml) as independent variables. The slope of the standard curves for the nine AHSV reference strains in all optimised virus-species- and serotype-specific assays, were then used to estimate the efficiency of the individual assays in detection of each of the reference strain. The efficiency was calculated by the formula 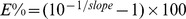. The slopes values of the nine reference strains in the individual assays were than compared to the respective common regression line using analysis of variance (ANOVA) procedure. All statistical analyses were performed and graphs were plotted in R 2.15.2 [70].

Efficiencies of Seg-1 and Seg-3 assays were also estimated on the basis of standard curves plotting Ct values against corresponding log dsRNA copy number per reaction.

## Results

### Sequence Comparison and Assay Design

#### Seg-1 and Seg-3 based (serogroup/virus-species-specific) assays

Comparisons of Seg-1 or Seg-3 nucleotide sequences, from the nine reference strains of AHSV, along with publicly available sequence data (Table 1), identified conserved regions suitable for the development of virus-species -specific RT-PCR assays (Figure S1 in File S1 and Table 2). Some redundant/ambiguous bases were introduced within the Seg-1 probe and Seg-3 forward and reverse primers, to accommodate nucleotide differences between individual virus strains (Table 2) and ensure assay sensitivity and specificity. Although some differences in the target-footprints still remain in some of the isolates, compared to the primers and probes, these were shown to have little impact on the specificity and sensitivity of the respective assays with the isolates used here for validation (Table 6).

**Table 6:** Diagnostic sensitivity of virus-species- (Seg-1 and Seg-3) and type-specific (Seg-2) assays.

AHSV isolate	virus-species -specific		AHSV type-specific assays								
	Seg-1	Seg-3	–1	–2	–3	–4	–5	–6	–7	–8	–9
**Reference strains**											
RSArah1/03	17.36	19.13	18.39	No Ct	No Ct	No Ct	No Ct	No Ct	No Ct	No Ct	No Ct
RSArah2/03	16.56	16.02	No Ct	15.57	No Ct	No Ct	No Ct	No Ct	No Ct	No Ct	No Ct
RSArah3/03	21.10	22.02	No Ct	No Ct	21.18	No Ct	No Ct	No Ct	No Ct	No Ct	No Ct
SPArah4/03	14.37	16.09	No Ct	No Ct	No Ct	17.10	No Ct	No Ct	No Ct	No Ct	No Ct
RSArah5/03	19.02	20.52	No Ct	No Ct	No Ct	No Ct	22.67	No Ct	No Ct	No Ct	No Ct
RSArah6/03	20.00	19.81	No Ct	No Ct	No Ct	No Ct	No Ct	22.95	No Ct	No Ct	No Ct
KENrah7/03	17.22	16.17	No Ct	No Ct	No Ct	No Ct	No Ct	No Ct	19.24	No Ct	No Ct
RSArah8/03	17.08	16.41	No Ct	No Ct	No Ct	No Ct	No Ct	No Ct	No Ct	18.41	No Ct
PAKrah9/03	16.73	12.63	No Ct	No Ct	No Ct	No Ct	No Ct	No Ct	No Ct	No Ct	11.73
**Field strains**											
ETH2010/01	18.41	17.44	No Ct	15.51	No Ct	No Ct	No Ct	No Ct	No Ct	No Ct	No Ct
ETH2010/20	23.56	27.52	No Ct	25.26	No Ct	No Ct	No Ct	No Ct	No Ct	No Ct	No Ct
GHA2010/01	20.71	19.69	No Ct	20.09	No Ct	No Ct	No Ct	No Ct	No Ct	No Ct	No Ct
GHA2010/02	15.10	19.49	No Ct	21.48	No Ct	No Ct	No Ct	No Ct	No Ct	No Ct	No Ct
GHA2010/03	30.80	32.07	No Ct	32.09	No Ct	No Ct	No Ct	No Ct	No Ct	No Ct	No Ct
GHA2010/04	12.55	10.09	No Ct	12.49	No Ct	No Ct	No Ct	No Ct	No Ct	No Ct	No Ct
GHA2010/05	14.31	15.89	No Ct	15.57	No Ct	No Ct	No Ct	No Ct	No Ct	No Ct	No Ct
SEN2007/01	11.74	14.26	No Ct	15.47	No Ct	No Ct	No Ct	No Ct	No Ct	No Ct	No Ct
SEN2007/02	12.92	16.86	No Ct	15.72	No Ct	No Ct	No Ct	No Ct	No Ct	No Ct	No Ct
SEN2007/03	28.19	31.46	No Ct	28.46	No Ct	No Ct	No Ct	No Ct	No Ct	No Ct	No Ct
SEN2007/04	27.20	29.28	No Ct	21.54	No Ct	No Ct	No Ct	No Ct	No Ct	No Ct	No Ct
SEN2007/05	20.59	23.06	No Ct	17.59	No Ct	No Ct	No Ct	No Ct	No Ct	No Ct	No Ct
ETH2010/03	16.82	14.24	No Ct	No Ct	No Ct	12.57	No Ct	No Ct	No Ct	No Ct	No Ct
ETH2010/04	13.43	9.88	No Ct	No Ct	No Ct	7.78	No Ct	No Ct	No Ct	No Ct	No Ct
ETH2010/05	17.80	16.91	No Ct	No Ct	No Ct	12.20	No Ct	No Ct	No Ct	No Ct	No Ct
ETH2010/06	19.40	15.80	No Ct	No Ct	No Ct	14.67	No Ct	No Ct	No Ct	No Ct	No Ct
ETH2010/07	19.44	20.62	No Ct	No Ct	No Ct	25.07	No Ct	No Ct	No Ct	No Ct	No Ct
KEN2007/01	20.17	20.91	No Ct	No Ct	No Ct	22.30	No Ct	No Ct	No Ct	No Ct	No Ct
SPA1987/01	12.36	15.93	No Ct	No Ct	No Ct	18.46	No Ct	No Ct	No Ct	No Ct	No Ct
ETH2010/19	15.97	22.39	No Ct	No Ct	No Ct	No Ct	No Ct	23.90	No Ct	No Ct	No Ct
SEN2007/06	14.77	19.09	No Ct	No Ct	No Ct	No Ct	No Ct	No Ct	24.56	No Ct	No Ct
ETH2010/10	23.61	21.59	No Ct	No Ct	No Ct	No Ct	No Ct	No Ct	No Ct	22.82	No Ct
ETH2010/11	18.31	18.84	No Ct	No Ct	No Ct	No Ct	No Ct	No Ct	No Ct	19.04	No Ct
ETH2010/14	21.05	24.69	No Ct	No Ct	No Ct	No Ct	No Ct	No Ct	No Ct	No Ct	25.41
ETH2010/15	21.95	22.46	No Ct	No Ct	No Ct	No Ct	No Ct	No Ct	No Ct	No Ct	28.79
ETH2010/16	20.47	21.53	No Ct	No Ct	No Ct	No Ct	No Ct	No Ct	No Ct	No Ct	21.17
GAM2009/01	14.48	17.39	No Ct	No Ct	No Ct	No Ct	No Ct	No Ct	No Ct	No Ct	20.15
GAM2009/02	14.16	15.89	No Ct	No Ct	No Ct	No Ct	No Ct	No Ct	No Ct	No Ct	18.59
KEN2006/01	15.89	25.86	No Ct	No Ct	No Ct	No Ct	No Ct	No Ct	No Ct	No Ct	20.27
**Vaccine strains**											
ETH2010/02	11.62	10.80	No Ct	27.42	17.27	No Ct	No Ct	No Ct	No Ct	No Ct	No Ct
ETH2010/09	21.11	20.32	No Ct	No Ct	No Ct	No Ct	24.39	No Ct	29.20	No Ct	No Ct
ETH2010/18	6.90	9.33	No Ct	No Ct	No Ct	No Ct	11.24	No Ct	No Ct	No Ct	23.04
SENvvv1/09	18.87	17.98	No Ct	No Ct	No Ct	No Ct	No Ct	No Ct	No Ct	No Ct	19.16

The Ct values obtained for homologous serotypes are boxed in.

#### Seg-2 based assays

Publicly available and newly generated nucleotide sequences for Seg-2 of the nine AHSV serotypes, were analysed and compared (Table 1 and Figure S1 in File S1) to identify regions unique to each of the virus types (Table 2). Type-specific primers and probes were subsequently designed targeting these regions for each serotype (Figure S1 in File S1).

### Assay Specificity

#### Seg-1 and Seg-3 based assays

A panel of nine monotypic AHSV reference strains, representing the nine known serotypes of AHSV (Table 3) were tested in duplicate with both of the virus-species-specific assays, targeting Seg-1 or Seg-3. The resulting mean Ct values for most of the strains tested in the Seg-1 assay were similar to those obtained in Seg-3 assay, indicating that these tests detect viral RNA with similar efficiencies (Table 6).

The specificity of the AHSV virus-species-specific assays was further evaluated using 33 different field and vaccine strains of AHSV serotypes 2, 3, 4, 6, 7, 8, and 9, collected from different geographical locations in central Africa (Table 3). All of the isolates were detected (produced amplification) in both assays (Table 6). Most of the isolates tested (17/33) were detected with similar sensitivity (within 2 Ct value difference). While the Seg-1 assay amplified the RNA target earlier (lower Ct) in thirteen isolates, the Seg-3 based assay was more efficient in amplification (lower Ct) with RNA samples from the three Ethiopian isolates of AHSV-4. These variations suggest minor differences in the ‘target’ nucleotide sequences for primers or probes in Seg-1 and Seg-3, between the isolates tested.

“No Ct” results, were consistently generated with nucleic acid of *C. sonorensis*, uninfected horse bloods or cell culture supernatants (KC cells, BHK and Vero cells), or dsRNA from other closely related *Orbivirus* species (EEV, PHSV, EHDV and BTV) (Table 5). *In silico* analysis of Seg-1 and 3 sequence data for 22 *Orbivirus* species [64], indicated that neither primers nor probes of the AHSV virus-species-specific assays, would bind to, or amplify their RNA.

#### Seg-2 based (type-specific) assays

The RNAs of the nine monotypic AHSV reference strains were also tested with ‘typing-assays’ targeting Seg-2 of the different AHSV serotypes (Table 3). In each case amplification was only observed with the homologous assay (Table 6). Further testing using field strains of AHSV serotypes 2, 4, 6, 7, 8 and 9, only produced positive signals with the homologous serotype (Table 6). These RT-PCR results were confirmed by sequencing and phylogenetic analyses of Seg-2 for majority of the isolates tested (Table 3).

The AHSV-9 vaccine strain produced by the National Veterinary Research Institute, in Senegal (SENvvv1/09), and the three vaccines (AHSV-2, AHSV-7 and AHSV-9) manufactured at the National Veterinary Institute, Ethiopia (ETH2010/02, ETH2010/09 and ETH2010/18, respectively), were also tested using the real-time RT-PCR typing assays. Although, positive results were obtained in each case with the assays for the homologous serotype, heterologous types were also detected (Table 6). The AHSV-2 vaccine strain (ETH2010/02) also contained AHSV-3; the AHSV-7 vaccine strain (ETH2010/09) also contained AHSV-5; and the AHSV-9 vaccine strain (ETH2010/18) contained AHSV-5. These real-time RT-PCR data were confirmed by Seg-2 sequencing of the mixed Ethiopian vaccine strains and two distinctive consensus sequences were generated in each case. Comparison of the sequence data to the Seg-2 dataset of the nine AHSV reference strains, confirmed the real-time RT-PCR typing results.

Seg-2 from ETH2010/02 (the ‘AHSV-2’ vaccine) showed 96% and 99.7% nucleotide identities with RSArrah/02 (AHSV-2) and RSArrah/03 (AHSV-3) respectively, suggesting that it had been contaminated with the AHSV-3 reference strain, or a very similar virus.

Seg-2 of ETH2010/09 (‘AHSV-7’ vaccine) showed 99% and 98.1% nucleotide identities with KENrrah/07 (AHSV-7) and RSArrah/05 (AHSV-5) respectively, indicating contamination with an AHSV-5 that is related to but may be distinct from the reference strain.

Seg-2 of ETH2010/18 (AHSV-9 vaccine) showed 96.6% and 99.4% nucleotide identities with PAKrrah/09 (AHSV-9) and RSArrah/05 (AHSV-5), again indicating contamination with the AHSV-5 reference strain or a very similar virus strain. The close relationship of the ‘second’ strains detected in each of these vaccines to the reference strains suggests laboratory derived contamination.

Nucleic acid preparations derived from the uninfected host species (horse blood and *C. sonorensis*) or uninfected cell culture supernatants (KC cells, BHK cells and Vero cells) gave ‘no Ct’ values in any of the nine type-specific assays.

### Assay Sensitivity and Efficiency

#### Seg-1 and Seg-3 based (virus-species-specific) assays

**Figure 1:**
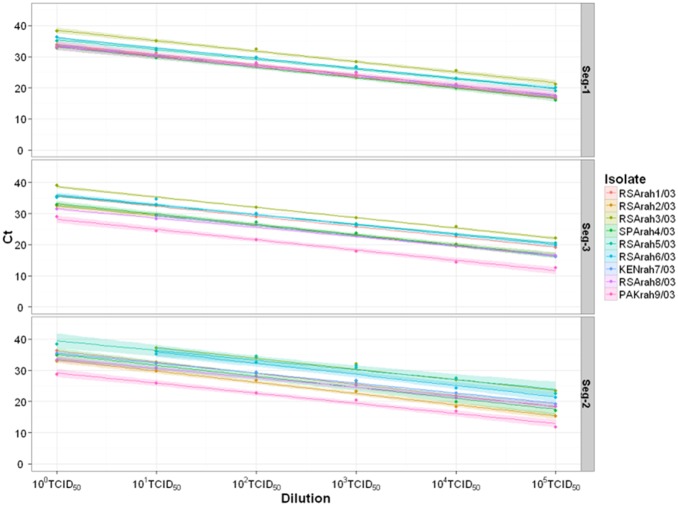
Standard curves for Ct value against virus titre for AHSV virus-species-and type-specific RT-PCR assays. Comparison of standard curves generated by plotting Ct values against RNA extracted from log virus dilutions with corresponding 95% confidence intervals (CI). All nine monotypic reference strains were analysed for each virus-species-specific assay while the homologous strains were analysed for each of the type-specific assays.

The efficiency rates of both virus-species-specific assays (Seg-1 and Seg-3) were calculated based on a dilution series of infectious virus, showing variations between 98% to 108%, or 102% to 115% respectively depending on the reference strain used (Table S1 in File S1). The slope, which is a measure of individual assay efficiency, showed only minor variations in either assay (from −3.0 to −3.4 with a good linear correlation (*R^2^*>0.98) for all AHSV types (Table S1 in File S1; Figure 1). No significant difference (p<0.05) was reported between the individual slopes in either assay by ANOVA test (Figure S2 in File S1).

**Figure 2:**
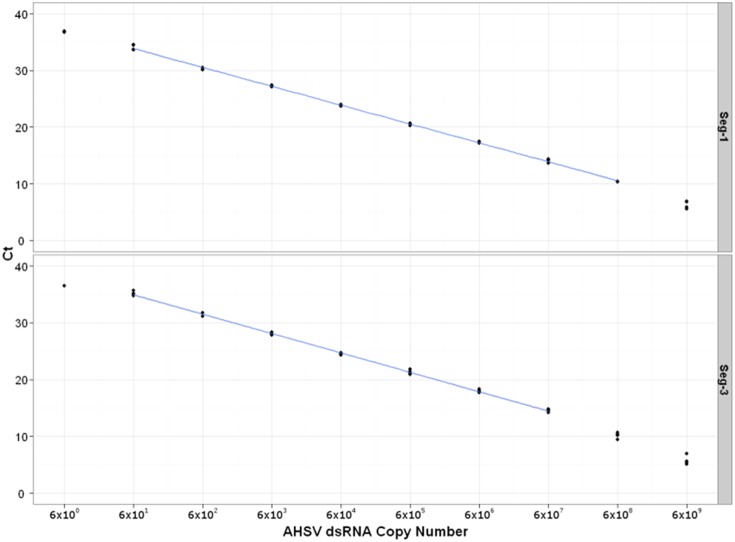
Standard curves for Ct value against RNA copy number for Seg-1 and Seg-3 RT-PCR. Standard curves generated by plotting Ct values against corresponding log copy number of viral dsRNA (RSArah01/03) in Seg-1 and Seg-3 assays. Each dilution was tested in quadruplicate. Non-linearity of the lowest and highest dilutions were excluded from the analysis.

Efficiency rates for the Seg-1 and Seg-3 assays were also calculated, based on a dilution series of viral RNA purified from AHSV-1 (RSArah1/03), showing values of 101% and 96% efficiency, estimated over eight (10^1^–10^8^) and seven (10^1^–10^7^) log dilutions respectively. Over these dilution ranges a linear dynamic with *R^2^* exceeding 0.99 was observed for both of the assays (Table S2 in File S1; Figure 2).

All nine serotypes of AHSV were detected with both virus-species-specific assays (Seg-1 and Seg-3) down to 1 TCID_50_/ml (since each PCR reaction contains RNA extracted from 0.029 ml of sample, this is equivalent to 0.029 TCID_50_ detected per assay). For AHSV-1 (RSArah1/03) this gave a Ct value of 33.77 in the Seg-1 specific assay, and 35.46 in the Seg-3 specific assay (Table S1 in File S1, Figure 1). This suggests that virus isolation would become increasingly difficult for samples showing Ct values higher than 33 or 35 respectively in these assays.

The assays targeting Seg-1 and Seg-3 consistently detected 60 copies of AHSV-1 (RSArah1/03) RNA per reaction in four independent repeats, with average Ct values of 33.96 in the Seg-1 specific assay and 35.16 in the Seg-3 specific assay (Table S2 in File S1). However, at 6 copies per reaction, the viral RNA was only detected in two of the four repeats in the Seg-1 assay, and one out of four in the Seg-3 assay. Based on the observation that they produce similar Ct values (see above), 60 copies of the viral genome per reaction appears to be approximately equivalent to 0.029 TCID_50_ (representing 2068 genome copies per ml, or 1 TCID_50_/ml of the original virus sample).

#### Seg-2 based assays (serotyping assays)

The RNA of the homologous AHSV serotype was detected by eight of the ‘typing’ assays, at all five dilutions down to 1 TCID_50_/ml (0.019 TCID_50_/assay). However, the AHSV-6 specific assay only gave consistent positive results down to 10 TCID_50_/ml (0.19 TCID_50_/assay) (Table S1 in File S1).

Efficiency rates calculated for the different typing assays, on the basis of the dilutions series of infectious virus, varied between 90–113% (Table S1 in File S1) reflected by a range of slope values (between −3 and −3.6). All nine of the Seg-2 assays showed linearity (*R* ^2^>0.98) (Table S1 in File S1; Figure 1). No statistical difference was identified between slopes using ANOVA analysis (p<0.05) (Figure S2 in File S1).

## Discussion

Real-time methods offer many advantages over conventional RT-PCR for the detection of viral RNAs. This includes high sensitivity and specificity, the ability to quantify viral RNA templates (copy number) and the detection of short amplicons, reducing potential problems caused by partial degradation of the RNA target. The reactions and detection also take place in a closed tube, reducing risks of laboratory based cross-contamination and false positive results. In addition, automation can be used to increase throughput and reproducibility. However, the very high specificity of real-time RT-PCR primers and probes can also lead to false negative results [72]. The most conserved genome segments or regions, that are also unique to the target virus-species, should therefore be selected for the design of real-time RT-PCR assays and a wide selection of diverse samples (including field and historical isolates) should be used for assay validation.

We report the development of real-time RT-PCR assays based on TaqMan technology for detection, identification and typing of AHSV RNA extracted from diagnostic samples, insects or infected tissue cultures. The typing assays targeting Seg-2 were validated alongside virus-species-specific assays detecting highly conserved regions of Seg-1 and Seg-3. These assays are considerably more sensitive than conventional RT-PCR assays previously published for typing AHSV RNA [57], [58]. The combination of virus-species - and type-specific diagnostic systems provides a complete set of tools for rapid and accurate detection and identification of AHSV. Both assays targeting Seg-1 and Seg-3 were shown to be entirely specific for AHSV. There was also no evidence for cross-amplification of RNA from heterologous serotypes by any of the type-specific assays (Seg-2).

All of the assays detected a wide range of reference, field and vaccine strains confirming their diagnostic sensitivity and specificity, indicating that they can be used for molecular diagnosis, surveillance and molecular epidemiology studies of AHSV. Some AHSV isolates were detected with different sensitivities by assays targeting Seg-1 and Seg-3, suggesting minor differences in the primer binding-regions, between these virus isolates. Both of the virus-species-specific assays were designed to target highly conserved regions of the most-conserved genome-segments (encoding structural proteins VP1 and VP3 respectively). However, it is possible that novel strains of AHSV will arise in the field that have further changes in these target ‘footprints’, significantly reducing the efficiency of one or other of these assays. This would require a ‘redesign’ of the assay to maintain its effectiveness. It is considered unlikely that both virus-species-specific assays would fail simultaneously and their use in tandem would maintain diagnostic capability reducing the risk of false negatives.

AHSV infection generates a relatively low level of viraemia in horses (up to ∼10^5^ TCID_50_/ml) and an even lower circulating titre in donkeys and zebras (<10^3^ TCID_50_/ml) [73]. Studies of analytical sensitivity reported here for the RT-PCR assays, indicate that a viraemic animal would be detected reliably using either the virus-species-specific or type-specific assays.

Both the virus-species- and type-specific assays are highly efficient, doubling amplicon quantity during each round of amplification in the geometric phase of the reaction (Figure 1 and 2). The virus-species-specific assays (Seg-1 and Seg-3) can be used to quantify the amounts of viral dsRNA, even at very low concentrations (down to 60 copies of viral dsRNA per reaction) (Figure 2). These assays were also considerably more sensitive than infectivity tests (∼2000 fold) indicating that (depending on the volume of inoculum used) a Ct of<33–35 would be needed before virus isolation is likely to be successful in cell culture.

Detection of AHSV RNA by RT-PCR assay is limited by the sample size that can be added to a single PCR reaction. However, our ability to isolate AHSV in cell cultures depends on the absence of neutralising antibodies, the level of virus-adaptation to cell culture, the type of cells used (e.g. insect or mammalian), the volume of inoculum, and the storage history (level of degradation of the sample).

In previous studies of BTV infectivity in BHK cells it was found that ∼1000 copies of the disaggregated and purified virus particle were equivalent to 1 TCID_50_ [71]. The lower infectivity to genome-copy ratio calculated here (2068 genome copies per TCID_50_) may reflect RNA losses during extraction from the virus, or an aggregated state of the unpurified virus.

As previously demonstrated for BTV, real-time RT-PCR assays are the most sensitive and reliable methods (that are currently available) for orbivirus detection and typing [74]–[76]. The virus-species-specific assays reported here can detect similar levels of RNA to other published real-time AHSV detection methods [51], [54]–[56]. However, the relatively small number of AHSV isolates that were available for this study (n = 42), especially for serotypes 1, 3 and 5, has implications for the wider validation of these assays, particularly for the type-specific (Seg-2) based assays. The diagnostic specificity of the nine typing assays described here, relates primarily to their inability to detect Seg-2 of non-homologous types, while still detecting all available isolates from the homologous AHSV type. If a novel isolate of the virus is identified (e.g. by the virus-species-specific assays) that fails to amplify using the typing assays, it should be sequenced (Seg-2) to provide a basis for further development/refinement of the relevant primers and probes. Similar problems have been addressed with conventional and real-time RT-PCR assays detecting Seg-2 of BTV, in order to maintain their specificity and sensitivity [77].

The assays described here are currently being used for diagnostic and/or research purposes in a wide range of host and insect vector species, by the reference laboratories and research groups at The Pirbright Institute. The Seg-1 based virus-species-specific assay was used to detect AHSV-4 dsRNA in blood from IFNAR-/- mice [37], while replication of AHSV-4 in orally infected *C. sonorensis* was assessed using the Seg-3 specific assay [78]. The AHSV types which caused recent multi-serotype outbreaks in Ethiopia (2010) [22] were characterised using the Seg-2 based typing assays and results were confirmed by sequencing. The protocol for all of the assays has been optimised to maximise sensitivity and uses the same thermal profile, allowing multiple assays to proceed on a single machine at the same time.

In conclusion, these studies have provided an easy to use ‘TaqMan’ real-time RT-PCR based methods, which represent fast, robust and reliable tools for the detection and identification (typing) of AHSV. They provide a basis for the design and timely implementation of control measures for AHSV, including vaccination programmes. These diagnostic methods (particularly if linked to sequencing and phylogenetic studies) could be used for epidemiology studies to improve our understanding of the prevalence, distribution and movements of specific AHSV strains.

## Supporting Information

File S1 **Figures S1–S2 and Tables S1–S2.** Table S1: Analytical sensitivity and efficiency of virus-species-specific (Seg-1 and Seg-3) and type-specific (Seg-2) assays with serially diluted infectious virus. Table S2: Analytical sensitivity and efficiency of virus-species-specific (Seg-1 and Seg-3) assays, with serially diluted dsRNA standards (RSArrah/01). Figure S1: Nucleotide alignments for the design of AHSV virus-species (Seg-1 and Seg-3) and type-specific (Seg-2) assays. Seg-1 and Seg-3 indicate the virus-species-specific assays while SRT-1 to SRT-9 indicate respective type-specific assays. The nucleotide sequence of primers and probes for individual assays, including redundant bases, are listed in Table 2. Figure S2: Slopes values in relation to the slope of the common regression line for virus-species and type-specific RT-PCR assays. Comparison of slopes of the nine reference strains to the respective common regression line in virus-species-specific (Seg-1 and Seg-3) and type-specific (Seg-2) assays with corresponding 95% CI. Slopes of all nine serotypes of AHSV are highly similar in each of the virus-species-specific or individual type-specific assays respectively. Solid red line represents common regression line while dotted lines mark defines the 95% CI.
(DOC)
