# Correction: Real Time RT-PCR Assays for Detection and Typing of African Horse Sickness Virus

**DOI:** 10.1371/journal.pone.0104665

**Published:** 2014-08-01

**Authors:** The PLOS ONE Staff

There is an error in Table 1. Accession number KF446257 listed for Seg-3 of AHSV-3 should be KF446258. Please see the correct table below.

**Table 1:** Sequence data used to design real time RT-PCR assays.

Virus serotype	Origin	ORC reference number 2	Seg-1 Acc. number	Seg-2 Acc. number	Seg-3 Acc. number
**Reference strains** 1					
AHSV-1	Republic of South Africa	RSArrah/01*	KF446265	KF446274	KF446256
AHSV-2	Republic of South Africa	RSArrah/02*	KF446266	KF446275	KF446257
AHSV-3	Republic of South Africa	RSArrah/03*	KF446267	KF446276	KF446258
AHSV-4	Spain	SPArrah/04*	KF446268	KF446277	KF446259
AHSV-5	Republic of South Africa	RSArrah/05*	KF446269	KF446278	KF446260
AHSV-6	Republic of South Africa	RSArrah/06*	KF446270	KF446279	KF446261
AHSV-7	Kenya	KENrrah/07*	KF446271	KF446280	KF446262
AHSV-8	Republic of South Africa	RSArrah/08*	KF446272	KF446281	KF446263
AHSV-9	Pakistan	PAKrrah/09*	KF446273	KF446282	KF446264
**Sequence data from international databases**					
AHSV-1			NC006021 FJ011107 FJ183364 AM883164	AY163329 FJ011108 AM883165	AM883166 FJ011109 EU303138
AHSV-2			FJ196584	AY163332 FJ196585	EU303139
AHSV-3				DQ868772 U01832 Z26316	EU303136 EU303135 EU303134 EU303133 EU303132 EU303140
AHSV-4				EU046574 DQ868773 U21956	EU303141 EU303137 D26572
AHSV-5				AY163331	
AHSV-6				DQ868774 AF021235	EU303142 AF021236
AHSV-7				DQ118706 DQ118705 DQ118704 DQ118703 AY159954 AY159953 AY159952 AY159951 AY159950 AY159949 AY159948 AY159947 AY159946 AY159945 AY159944 AY159943 AY159942 AY159941 AY159940 AY163330	
AHSV-8				DQ868775 AY163333	EU303143
AHSV-9			U94887	DQ868776 AF043926	

* Isolates sequenced as part of this study.

1 Set of nine monotypic AHSV reference strains (Bachanek-Bankowska et al – in preparation)

2 Orbivirus reference collection (ORC), The Pirbright Institute.
