# Supplementary material for: Real Time RT-PCR Assays for Detection and Typing of African Horse Sickness Virus
Source: PLoS One. 2014 Apr 10;9(4):e93758. doi: 10.1371/journal.pone.0093758 (PMC3983086; doi:10.1371/journal.pone.0093758)
Supplement: File S1 — Figures S1–S2 and Tables S1–S2. Table S1: Analytical sensitivity and efficiency of virus-species-specific (Seg-1 and Seg-3) and type-specific (Seg-2) assays with serially diluted infectious virus. Table S2: Analytical sensitivity and efficiency of virus-species-specific (Seg-1 and Seg-3) assays, with serially diluted dsRNA standards (RSArrah/01). Figure S1: Nucleotide alignments for the design of AHSV virus-species (Seg-1 and Seg-3) and type-specific (Seg-2) assays. Seg-1 and Seg-3 indicate the virus-species-specific assays while SRT-1 to SRT-9 indicate respective type-specific assays. The nucleotide sequence of primers and probes for individual assays, including redundant bases, are listed in Table 2. Figure S2: Slopes values in relation to the slope of the common regression line for virus-species and type-specific RT-PCR assays. Comparison of slopes of the nine reference strains to the respective common regression line in virus-species-specific (Seg-1 and Seg-3) and type-specific (Seg-2) assays with corresponding 95% CI. Slopes of all nine serotypes of AHSV are highly similar in each of the virus-species-specific or individual type-specific assays respectively. Solid red line represents common regression line while dotted lines mark defines the 95% CI. (DOC) [file pone.0093758.s001.doc]

Table S1: Analytical sensitivity and efficiency of virus-species-specific (Seg-1 and Seg-3) and type-specific (Seg-2) assays with serially diluted infectious virus.

| **Assay** | **Isolate** | **Slope** | **Efficiency (%)** | **R^2^** | **TCID_50_/ml** | | | | | |
| --- | --- | --- | --- | --- | --- | --- | --- | --- | --- | --- |
|  |  |  |  |  | **10^5^** | **10^4^** | **10^3^** | **10^2^** | **10^1^** | **10^0^** |
| Seg-1 | RSArah1/03 | -3.296 | 101.1 | 0.998 | 17.36 | 21.06 | 24.17 | 27.38 | 31.09 | 33.77 |
| Seg-1 | RSArah2/03 | -3.346 | 99.0 | 0.997 | 16.56 | 20.65 | 23.17 | 26.89 | 29.83 | 33.73 |
| Seg-1 | RSArah3/03 | -3.361 | 98.4 | 0.994 | 21.10 | 25.53 | 28.30 | 32.41 | 35.00 | 38.12 |
| Seg-1 | RSArah4/03 | -3.303 | 100.8 | 0.993 | 16.07 | 19.91 | 23.71 | 27.20 | 29.51 | 32.73 |
| Seg-1 | RSArah5/03 | -3.138 | 108.3 | 0.991 | 19.02 | 23.09 | 26.69 | 29.66 | 32.06 | 35.01 |
| Seg-1 | RSArah6/03 | -3.243 | 103.4 | 0.999 | 20.00 | 22.90 | 26.37 | 29.62 | 32.59 | 36.24 |
| Seg-1 | KENrah7/03 | -3.218 | 104.5 | 0.992 | 17.22 | 20.41 | 24.91 | 27.66 | 30.30 | 33.26 |
| Seg-1 | RSArah8/03 | -3.228 | 104.1 | 0.999 | 17.08 | 20.37 | 23.53 | 27.08 | 30.13 | 33.11 |
| Seg-1 | PAKrah9/03 | -3.177 | 106.4 | 0.985 | 16.73 | 21.09 | 24.77 | 28.04 | 30.08 | 32.92 |
| Seg-3 | RSArah1/03 | -3.265 | 102.4 | 0.999 | 19.13 | 22.63 | 25.85 | 28.90 | 32.49 | 35.46 |
| Seg-3 | RSArah2/03 | -3.192 | 105.7 | 0.995 | 16.02 | 20.23 | 23.56 | 26.29 | 29.06 | 32.52 |
| Seg-3 | RSArah3/03 | -3.284 | 101.6 | 0.995 | 22.02 | 25.71 | 28.54 | 31.91 | 34.55 | 39.03 |
| Seg-3 | RSArah4/03 | -3.304 | 100.7 | 0.993 | 16.09 | 19.93 | 23.73 | 27.23 | 29.53 | 32.76 |
| Seg-3 | RSArah5/03 | -3.055 | 112.5 | 0.998 | 20.52 | 23.18 | 26.74 | 29.87 | 32.86 | 35.47 |
| Seg-3 | RSArah6/03 | -3.282 | 101.7 | 0.979 | 19.81 | 23.30 | 26.12 | 30.01 | 34.63 | 35.21 |
| Seg-3 | KENrah7/03 | -3.244 | 103.4 | 0.999 | 16.17 | 19.77 | 22.89 | 26.29 | 29.13 | 37.90* |
| Seg-3 | RSArah8/03 | -3.013 | 114.8 | 0.994 | 16.41 | 19.41 | 22.79 | 26.40 | 28.22 | 31.49 |
| Seg-3 | PAKrah9/03 | -3.297 | 101.1 | 0.985 | 12.63 | 14.26 | 17.88 | 21.62 | 24.41 | 28.87 |
| Seg-2 | RSArah1/03 | -3.587 | 90.0 | 0.999 | 18.30 | 21.83 | 25.82 | 29.06 | 32.58 | 36.31 |
| Seg-2 | RSArah2/03 | -3.598 | 89.7 | 0.994 | 15.27 | 18.38 | 23.24 | 26.72 | 29.71 | 32.96 |
| Seg-2 | RSArah3/03 | -3.432 | 95.6 | 0.982 | 23.44 | 26.95 | 31.99 | 33.99 | 37.08 | 34.97* |
| Seg-2 | RSArah4/03 | -3.529 | 92.0 | 0.967 | 17.10 | 19.95 | 26.61 | 29.02 | 30.65 | 34.90 |
| Seg-2 | RSArah5/03 | -3.111 | 109.6 | 0.962 | 22.48 | 27.43 | 31.46 | 34.53 | 36.03 | 38.48 |
| Seg-2 | RSArah6/03 | -3.595 | 89.7 | 0.961 | 21.34 | 24.31 | 30.73 | 32.58 | 35.18 | No Ct |
| Seg-2 | KENrah7/03 | -3.196 | 105.5 | 0.995 | 19.24 | 22.69 | 26.78 | 29.34 | 32.24 | 35.37 |
| Seg-2 | RSArah8/03 | -3.041 | 113.2 | 0.993 | 18.41 | 21.05 | 25.12 | 28.13 | 30.86 | 33.21 |
| Seg-2 | PAKrah9/03 | -3.252 | 103.0 | 0.982 | 11.73 | 16.90 | 20.50 | 22.73 | 25.83 | 28.69 |

Both group-specific assays (Seg-1 and Seg-2) detected all nine serotypes of AHSV diluted to down to 1 TCID_50_/ml (last dilution analysed). Similarly most of the type-specific assays also detected the homologous AHSV type diluted to down to 1 TCID_50_/ml (last dilution analysed) apart from the AHSV-6 assay which could detect the virus down to 10 TCID_50_/ml. The slope of the linear regression, its correlation (*R*^2^) and efficiency were also calculated for each virus serotype in each assay. Values marked with asterisk (*) were not used for the calculations.

Table S2: Analytical sensitivity and efficiency of virus-species-specific (Seg-1 and Seg-3) assays, with serially diluted dsRNA standards (RSArrah/01).

| **Assay** | **Isolate** | **Slope** | **Eff**  **(%)** | **R^2^** | **Copy number** | | | | | | | | |
| --- | --- | --- | --- | --- | --- | --- | --- | --- | --- | --- | --- | --- | --- |
|  |  |  |  |  | **6x10^8^** | **6x10^7^** | **6x10^6^** | **6x10^5^** | **6x10^4^** | **6x10^3^** | **6x10^2^** | **6x10^1^** | **6** |
| Seg-1 | RSArah01/03 | -3.3 | 100.9 | 0.998 | 10.37 | 14.06 | 17.39 | 20.54 | 23.83 | 27.23 | 30.31 | 33.96 | 37.89* |
| Seg-3 | RSArah01/03 | -3.415 | 96.3 | 0.998 | 10.14* | 14.49 | 17.98 | 21.27 | 24.58 | 28.01 | 31.40 | 35.14 | 39.01* |

Mean values of four repeats per each dilution are presented. dsRNA diluted down to 60 copies (equivalent to 60 virus particles) per reaction could be detected with 100% analytical sensitivity (in all four repeats), while a six copies of the virus per reaction were detected in 50% of the repeats (2 out of 4) in the Seg-1 assay and 25% (1 out of 4) in the Seg-3 assay. The slopes for each assay have been estimated and assay’s efficiency and correlation (*R*^2^) calculated. Values marked with asterisk (*) were not used for the calculations. Eff = Efficiency.

Virus-species-specific assays:

**Seg-1 assay**

1310 1320 1330 1340 1350 1360 1370 1380 1390 1400 1410 1420 1430 1440 1450 1460

..|....|....|....|....|....|....|....|....|....|....|....|....|....|....|....|....|....|....|....|....|....|....|....|....|....|....|....|....|....|....|....

AHSV-1 RSArrah/01 **TGCCTGAAGATATGTATACATCGATATTACGTCTTGCTAAAAACACAAGCTCAGGCTTTTCAACTAGTATTGATGTTTTTAAGCGGTATGGTCCCAACGCGAAAGGTGGACGTGGAGAAAAGATCCAAATAACTTCGCGGATTAAAGCGTTAGTGAT**

AHSV-2 RSArrah/02 **.............................................................................................................................................................**

AHSV-3 RSArrah/03 **................C.....A....................T.................T.........................................................................................G.....**

AHSV-4 SPArrah/04 **.............................................................................................................................................................**

ahsv-5 RSArrah/05 **....C....................T.................T...........T..C.......................A....................G....................T........G....................C..**

AHSV-6 RSArrah/06 **...................G.....T.................T........G..T..C.......................A.................A..G.............................G....................C..**

AHSV-7 KENrrah/07 **.............................................................................................................................................................**

AHSV-8 RSArrah/08 **....C....................T.................T........G..T..C.......................A....................G....................T........G....................C..**

AHSV-9 PAKrrah/09 **..........C................................T........G................................A........T.....A..G................................A........G...........**

AHSV-1 NC006021 **.........................T........C........T........G..T..C................................C...........G....................T.....T..G....................C..**

AHSV-1 FJ011107 **.............................................................................................................................................................**

AHSV-1 FJ183364 **.............................................................................................................................................................**

AHSV-1 AM883164 **.............................................................................................................................................................**

AHSV-2 FJ196584 **....C....................T.................T.....T..G..T..C.......................A....................G...........G........T........G....................C..**

AHSV-9 U94887 **.........................T........C........T........G..T..C................................C...........G....................T.....T..G....................C..**

**Seg-3 assay**

2030 2040 2050 2060 2070 2080 2090 2100 2110 2120 2130 2140

....|....|....|....|....|....|....|....|....|....|....|....|....|....|....|....|....|....|....|....|....|....|....|...

AHSV-1 RSArrah/01 **TACGAGATATTGTAAGGTGGAGTCAACAGAGGGACATTCAGGAGTCGTTGGCGTATGTTTTGAATAGGGAAGCTTGGGCGATTGCTAATGACTTTGAAGACTTGATGTTAGTGGATCA**

AHSV-2 RSArrah/02 **..................................T..............A..A.....C.........................................T...........A.....**

AHSV-3 RSArrah/03 **.........................G........T..............A..A.....C.........................................T.................**

AHSV-4 SPArrah/04 **.............G...................................A..A.....C................................T........T.................**

AHSV-5 RSArrah/05 **.............G...................................A..A.....C................................T........T.................**

AHSV-6 RSArrah/06 **.G........C......................................A........C.........................................T..............C..**

AHSV-7 KENrrah/07 **.................................................A....................................................................**

AHSV-8 RSArrah/08 **.............G...................................A..A.....C................................T........T.................**

AHSV-9 PAKrrah/09 **.................................................A........C.......................C........T........T..............C..**

AHSV-1 AM883166 **......................................................................................................................**

AHSV-1 FJ011109 **......................................................................................................................**

AHSV-1 EU303138 **......................................................................................................................**

AHSV-2 EU303139 **..................................T..............A..A.....C.........................................T...........A.....**

AHSV-3 EU303136 **......................................................................................................................**

AHSV-3 EU303135 **......................................................................................................................**

AHSV-3 EU303134 **......................................................................................................................**

AHSV-3 EU303133 **......................................................................................................................**

AHSV-3 EU303132 **......................................................................................................................**

AHSV-3 EU303140 **.........................G........T..............A..A.....C.........................................T.................**

AHSV-4 EU303141 **.................................................A....................................................................**

AHSV-4 EU303137 **..................................T..............A..A.....C.............................C...........T.................**

AHSV-4 D26572 **.................................................A..A.....C.......................C.................T......C..........**

AHSV-6 EU303142 **..................................T..............A..A.....C.............................C...........T.................**

AHSV-6 AF021236 **.G........C......................................A........C.........................................T..............C..**

AHSV-8 EU303143 **..................................T..............A..A.....C..........................C..............T.................**

Type-specific assays (Seg-2):

**SRT-1 assay**

AHSV-1 RSArrah/01 **ATCCGAATGGTAAGCTTTGGATTGAACATAAACAAACGGTGAGTGAGCAGTTGAAAAAGAAACAAGAGGAGAACCGAGCG---CCTCTAACCG**

AHSV-1 AY163329 **................................................................................---..........**

AHSV-1 FJ011108 **................................................................................---..........**

AHSV-1 AM883165 **................................................................................---..........**

AHSV-2 RSArrah/02 **........AAGGGTT.A....AC.....C.......AA....AGA.A..A..AG..G.A.....G..A.....TG.GT.T---..CA.GT...**

AHSV-3 RSArrah/03 **.C..A...AA.GGTACG....G.A.G......GCGGAA..T.AGA..TTT.....CG..G.....A..A....TGA.AATAAA..AT...AG.**

AHSV-4 SPArrah/04 **........AAGGGTAAG....AA.......T.A.GGA...A.CC..AA.A.....G..AGCG...ACC..A..TG...GACAA..ATGCCAA.**

AHSV-5 RSArrah/05 **....A..C......AA.....AA..TT.C..GA...A...ACAA.........G.TG..GCG...A..A.....AATAACGAA..GTAT.AA.**

AHSV-6 RSArrah/06 **........CACC..ACG....AG..T....TTA..GAA....C...T..AA....TCGTGCG..GC.A.GC...AATAATAAG.......AG.**

AHSV-7 KENrrah/07 **....T..CAA.TC.ACG.....C..G.....GA.G.A.A..GCG..AA..C.A..GG.TG.G...C..A.A....A.AATCGT..GA.GCTA.**

AHSV-8 RSArrah/08 **.......C..CG.AAA.....AAA..T....GGCGGAA..CCAA..A.GT.....TG.AGCG..GA..A....TG...GTGTT....ATTTAA**

AHSV-9 PAKrrah/09 **....A...CA.G.TACA....AAA.T...GTCA..GAAA.C..A...AG.A..C.G..AG....GAGC.C...TTCCAACTCA..CT...AGA**

**SRT-2 assay**

**AHSV-2 RSArrah/02** **GAGAAGTGGACTTCGATTATAGATGATCCTCAGTCCTACTTTGAGGATGATATATTTGCGGGAAAAGCTAATAGGATGTTTTTGAGAGGCGGAGAGACGGTTGAACGGCATGTGAAATTGAAGGTTAACGCTCAGACAGAGAACGT**

**AHSV-2 AY163332**  **..................................................................................................................................................**

**AHSV-2 FJ196585**  **..................................................................................................................................................**

**AHSV-1 RSArrah/01** **..A.......AGATT..CG.T........G..AA.T.....C..A.....CT.G..C.TT.ATCGG..G..C.A...T..C.CT..G..T...C.......GA.T.A..TGA.A.GTA.C.....G..T..G..AT..A.T.....**

**AHSV-3 RSArrah/03** **A..G.A....G.AAAGAGG.T.....C...G.AAAA.....C.TT......T...A.AATA..TGCC.GG.GTCA..A.A.G.C..GAA.....TTGATCC.A.TAA------T..GA.A.T.A....GAAG.GAGGTTTAGTT.G**

**AHSV-4 SPArrah/04** **CGA......GAGGATGAGG.T........AG..AGT..T...T.T......C.T.A.A.TAATG.GC.CC.C..AG.....C.A..C.CA...A..GAT..G..TAA------T..TA.C.C.C..CGAT.GATTT.----GCA.G**

**AHSV-5 RSArrah/05** **AG........GCGAT..AGCT......A.GG.AGAG..T...ATA......T.G.AC.TTAAT..GC.AG.....G.C...GA.....CT..GTTAGATCCG..GA.------AC.CA.C........A.G.GT..T----G.ATG**

**AHSV-6 RSArrah/06** **...C.T...GA.AA.G..G.G..A.....AG.A..T..T..CATA.....GC.T.A.CAAAAG..TC.GGCGGAAG......AT..C.CA..CT.TGATACA..T.A------A..TG..GTAA.CG.T.GGA.A.T----G..AG**

**AHSV-7 KENrrah/07** **A..G.....T.AAATGAGG....G..C..GG..GAT..T..C.TT...A.CT.G...AATAA..C.C.GG..GCAG.....GA.....A....AT.GAC.GAAGCAA------C.GGA.T.TC.....GAA.A.A..TACACTTCG**

**AHSV-8 RSArrah/08** **A.AG.A...GGAAA.G..TCT.....AA.GG.TGTT..T..CATC......T.....T.CTC..CGC.GG.C.A.G.A..CAGAC.T.TT...CTAGATTC.C..AA------T..TA.C...A..G.G.G.A.A.T----G.ATG**

**AHSV-9 PAKrrah/09** **...G.A...GAGAAA..CG.T.........G..AGG..T..CT.T.....GC.T...AAC.C.TC.C.AG.G.CAG.T..CA.T..CAAA..TT.TGACC....CAA------T..TA.CGTAA..G.G.GAA..GT----.GGTC**

**SRT-3 assay**

**AHSV-3 RSArrah/03** **ACAGCTAGAAAGAATGATGAGCAGTGAAGAGCTGGAAAGACCCGTTATTGAGAGCGTCATAAGATTCGGTTCTTTATATAACGCGCACGCGGGCAA-AAAGACAGGCGACATTCCATTAGAAGTCTTGATTAAAT**

**AHAV-3 DQ868772**  **...........................G.....A......................................C.......................-G................................A....**

**AHAV-3 U01832**  **........................................T.......................................................-......................................**

**AHAV-3 Z26316**  **........................................T.......................................................-......................................**

**AHSV-1 RSArrah/01** **CG..A.G.CT...TCA..TGAA.AC..T..AT.AAGCC.T.AA..GG....CCAAA.T...CA..AT...GGACA..T..GTT.ATG.T.A...GC-.CGTGA..AT..T.....GA..A.TA.T...T.GG.G.**

**AHSV-2 RSArrah/02** **GG..T.G.TG..TGCA..C.AAGAC..G.....AAGTC.GGGA..A....G.C.GA..G..CA..AT..C..GCA..T..GTT..TGT..A..GG.-.CGAGA..AT..T..C..GA.C...ACA......CGT.**

**AHSV-4 SPArrah/04** **TG.CT..CG.GTT.AA..TGAGC.G..T..TT.ATCTC..GAGACA...ATTCAGA....TGAG.A....AAGAA..T...TT.ATCA..A..TG.T..GC.GG..AACAT.-T..A.T...AAA...G.AG.G.**

**AHSV-5 RSArrah/05** **TACAT.GCGTGAGCGT..CGAG..G..T...T.A..TGA.AAA......C..GAAA....TGCT.AT..A..CAA..T..G.A.CT.......T.C-.CGC.....A..TC.CA.T..GA.T.AA..AG.A..G.**

**AHSV-6 RSArrah/06** **GACTT...CGGA.CGA...GAAGAG..G......ACTGAGGATA.....CGAGCA..G..C..G.AT..AG.CAA...CGCTA...G.T....T-.TGCGAGAG.ATACAC.GT..C.CC.G.AG...GA.CGT.**

**AHSV-7 KENrrah/07** **..G.T......A.C.....CAG.AA........T..G.ATT.T..G..C....AT..A...C.C.A......GCAG.T..G.A.A.....C..AG.-......TAAT..T..A..TC.GTCC..T.....A....**

**AHSV-8 RSArrah/08** **TGG.T..CGCGA.CGT..TGAA.AG..T...T....TGA.AAA......C.AGAGA.T..TGCC.AT..C..AAA..T..G.A.AT.TA.A..T.CC..GC..-..T..T..AT.GC..A..AA...AG..G.G.**

**AHSV-9 PAKrrah/09** **.A.CT.G.C...CCG....GAAGAC......T..AGTC.GGAAA.A..A.TTGCG..G..C.AT.AT.....GAAG.TCGGGA.TAGGT.C..G..-G..A.A..ATTTG..GA.CA.T..TAAGC.TGAA....**

**SRT-4 assay**

**AHSV-4 SPArrah/04** **CGTTTCGTATCATATTGGTATAGAATATCACAAGTTGAAGTAACGAAGGCGCGTAATGAAGTTCTGGACATGAATGAGAAACAGAAGCCGTATTTTGAATTTGAATATGATGATTTCAAACCCTGTTCAATTGGAGAGTTGGGGATCCATGCATCCACATATATATATCA**

**AHAV-4 EU046574**  **..........................................................................................................................................................................**

**AHAV-4 DQ868773**  **..........................................................................................................................................................................**

**AHAV-4 U21956**  **..............................................................................................................C..........A............................A...................**

**AHSV-1 RSArrah/01** **A.A..T..T..T.......TC......GA...TT..TCGA.T....CA..AAAA..GCG.AC.---.....TCGA..T...A...CTGAA...AAC..G.....C.TC..A..C.....G..GGCA.GT........A........A..C..G..G........C..C..**

**AHSV-2 RSArrah/02** **A.A..T.....T.......TC.....TGA..GCACAACGCACGTA....GAG.GGGAAG...A---..T..TCGG..TCG.A.A.CAGGA..CCAGC.G..C..TGTA..A.....T.....TGCAAGTG.A..G..AC.T..AT.T..C..G..A........T.....**

**AHSV-3 RSArrah/03** **A.A..T..C............GA.T.TCA.A.....ACTA.TGA.GCT.ATTCG..GCG.T.G---...GCACG..GCG....T.TA.A....CA...GA.A..TGT...A...........T..CG.G....CG..AC.T..AT.A...TGC..A..C.....T.....**

**AHSV-5 RSArrah/05** **......A.T..T..........A....A.TA.G.......C..G...TCTATTG.C.TT.AC.---..T..AGGG.G.G.TGCA..AAA....ACAC.G.....TCC.........T.....AATGG.TG.G.CT...C....AGCA..C..G..G..G...G.T.....**

**AHSV-6 RSArrah/06** **A.A..T..T..G.........C.TG.TGAGA..A.---.AC.......-----C.CCTTGAG.---T-TT.A.C...AG.GA.TCGAAAAGTCGCGC.G.....T.T...G....AT.....GATGG.T.........A.......T.....G..G.......AG...G.**

**AHSV-7 KENrrah/07** **.....T..T..G..........CGT.TGAGA.G..G..G..TGGTCG....ATGGCGAG.A.C---..T..TC....TCG.G...CCAA..T..CC..G..C..TGTC.....C.AT.....A......G.G.CG..AA.....C.A...T....A..T.....C.....**

**AHSV-8 RSArrah/08** **.....T..TA.C..........A...TA..AGG..G..GAAG.AAG.TTTA.TC.T..TGAA.---..T..CT.C..TG..A.A.C.GAA...CAGC.G.....TCCG...........G..AATGGTT.........A....TG.T..C..T..A..............**

**AHSV-9 PAKrrah/09** **..G..T...............C..G.TCG...G...C..ACCT.A...-----GGTATTGAGA---.-G.G.TCGATTG.GG.TGTCAAA...AGG..G.....TATA..AAG...T..G..T.ACG.T.....C...A.C..T..T..C.....A..T..C.AG.....**

**SRT-5 assay**

**AHSV-5 RSArrah/05** **AGAGACACATCAAGGTTAAAGGTGTGATGAA------TGAGCTGACGACGTACTTCTCAAAACGTTTCATTTCTTATTGGTATAAAATAACTAAGGTTGAAGCAAGGAATCTATTGACTTTAACTGAT---ATAGGGGGGGATGCAAAAAAGTATACACAGTTTGATCCTGAT**

**AHSV-5 AY163331**  **...............................------...........................................................................................---..........................................**

**AHSV-1 RSArrah/01** **T..T.AGT........G..T.CGCAATCA..TAACGT....GGT.....C..T..T..G...A.A..TG............TC.G....GAAC.TT..TCGATT.C..CAGC.AAA.AGCG......C---..TC.A.ATA.AAAG.CTG.A....ACG.......CTTC..A**

**AHSV-2 RSArrah/02** **.TGTGA.AT.G........C.C.CA..CAG.GAACGT....GGC.TC..A.....T......A.A..TG.A..........TC.G...TGAACGCACAACGCACGTA..GGG.GG.GGAAG.GTA...---..TC...ATCGAAA..C.GGA..CCAG.....C...GTA..A**

**AHSV-3 RSArrah/03** **.T.ATA.G..A.T.A....GAAGCGAGGTTTAGTTGGG...AGCCA.CG.C.T..T..TGCGA.A..TG.C..A.........G..T.TCAA..A...ACTATTGA.GC.GAT.C..AGCG.TTG..C---GC.C.T..C..ACAT.T.C.....CATG..A.A...GT...A**

**AHSV-4 SPArrah/04** **.T.ATA.T....C.C..CG.TCGA.TTC.C.------G.C.GAA..C.....TC.A..G.........G.A..A..........G....T.AC.A.......T..C...GGCGCGT.A.GA.GT.CTGGAC..GAAT.A.A.ACAG..GCC....TTTG.A.....ATA....**

**AHSV-6 RSArrah/06** **.TCA.A.TG.GGTAA.CG.T..GAAA....C------A...GGAGTC...........T...A.A..TG....G.........CGTG.TGAG..AA.---.A...C...G.---ACCT.GAGTT.TTA---.CT.AA.A.A..CG....---GTCG.G.........TT...G**

**AHSV-7 KENrrah/07** **GC.ACAGG..T.TC.....GAA.AAA.CT.CACTTCGA...GGACAACGCC.T..T...GCT.....TG....G..........CGT.TGAG.....G..G.TTG.TCGGGCGA..G.GAG..TC...---..TCAT.ATCGA.AG.CC....T.T.CG....C...GTC...**

**AHSV-8 RSArrah/08** **.A.ATA.T......A..G.G...AAA.....------....T....T..A.................TG..A.C..............T..A.G...G..GAAG.AAG..T..C.C.T.G.G.A....---..CTAC.AT..AAA..CGG.A...CAG...........G...**

**AHSV-9 PAKrrah/09** **.C.ATA.T...GTAA..G.G..AAA.G.AGG------.C..GATGTC..C..T..T..G.....G..TG.A..A.........CG.G.TCGAC.....C..A.CTCA..GG---GT.T.GAG.GGAGG---TCGATT.A.....TC...---....GGG........ATA..A**

**SRT-6 assay**

**AHSV-6 RSArrah/06** **CGTTGGTCATTGGGTCGATTGGGTTGTTGATTTGATTATGTTGGCGCAGGTGAAAATGTTAATAAAAGAATATAAGTTTAAAAGATTAAATAGCCAAAATTTGATGTCGGGTATGAACAAACTGGTTGGTGCGCTGAGATGTTATGCTTACTGCTTGATTCTTGCTTTATATGACTATTATGGTCAAGATATTGAGGGCTTCAAGAAGGGATCAAATTCGTC**

**AHSV-6 DQ868774**  **...............T..............C..A........A...........G................................G.............................T........C.....A.............................C.....G.......................C..........................T..**

**AHSV-6 AF021235**  **..............................................................................................................................................................................................................................**

**AHSV-6 NC_005996**  **..............................................................................................................................................................................................................................**

**AHSV-1 RSArrah/01** **A..A.A.........T..A.....C.....CACA..C...C.TAGAG..ACTG...AAA.G..T..G..T...CG...C....A.C.T..GC.TG.GG.GC....C..T..A.....T...T.A.AG.A..GCT.AC.....ATC.TG..T.....A..A......C....C..T.......AAGG......C.....T..T........GA.T.T.G.T..**

**AHSV-2 RSArrah/02** **G....A.........T..............CACA..C...C.TAA.G..ACTG.C.AAA.G.....G..T..CG.A.....G.A......AC.TG..G.A.....AG.T...........GT...AG.A..GTT.......CAT..T...T.........T.A..G........T........GGCT..G........A.....A..A..GA....CG.T..**

**AHSV-3 RSArrah/03** **...A.CG........G........C......A.CG.A...A..A.T..A..T.GCCGCA.G..............T....TT........A.AGG.TC.GC.A..CAGC........T..GT...AG.A..G.G.A.A...C..C..G..T..T.....C..C..AC.G..C..T.T.C....G.GT..GC.A..C.....TGC.C....GA.G.GAA.CG.**

**AHSV-4 SPArrah/04** **G...AA.......A.A........AACA...A.A......G.T.TA..AACT...CGT..GG.G.....G...GCA.......A.C....G...G....C..AC.TG.T..A.....T.GTT.A.....G.TAT.A.........ATG..T.....AGC.T.A..GA.C.....T.T.....AAGGGAC......T..T..T.....A..C..G...G.T..**

**AHSV-5 RSArrah/05** **.....AAA.C.................AA...AT........AT....C..C....G.C.GG.T..G..C.....A.....G..GC.C..GCCGG.T...........C..G.....T..G..A..C.....A..A...........G..T..T.....A..C..G........T....TC..GG.G.....C..A..G..T.....A...A.G..CG..G.**

**AHSV-7 KENrrah/07** **....ACA........T........C......A.T..A...ACAA.......TG...GAA.G........G...G.T......C....GG.G..AA..C.GC.A..TAGT...........G..C.AG.A..GAG.T.A............T.........T.G..A.....C..T........AG.T.CG..C.....T...GC.C.A...A.GCG.G..GG**

**AHSV-8 RSArrah/08** **....CAAA.......T........C..GA...AT..C.....AT....C......CGT...G.T..G..T.....A.......A.C.GC.ACCGG.T...........A..C.....T..G..T..A......T....G.....C..A..T...C.A..C.....G..G.....TC...TC..AGCG..A.....A..A..T.G......GA.T..CG.AG.**

**AHSV-9 PAKrrah/09** **G..C.AA......A.A.................A........A..A.....T...CGA.....T..G..G..C.......T.......G.G.CTACG..C..A...G.A..A.....T.....A..C..A..TT.AC.T..C.....C..T...........G...C.C.....T.TC......GCG.....C..A..T..TG.A.....T.....C..T..**

**SRT-7 assay**

**AHSV-7 KENrrah/07** **ATTCGACGTGGATCGAGCATAAGAAGAAGATGGCGGAAAAGCTAAAGGATGAGCAACAGAAAAACCAAAATCGTCCGATGCTAGTGCCGATTGATGGTGTCTACGTCTCCACTAGTGTTGAGTATGGTACCGTTACACATTGGGTTGATTGGGTCGTTGA**

**AHSV-7 DQ118706**  **.............................................................G.........................T........................A...............................................**

**AHSV-7 DQ118705**  **.............................................................G.........................T........................A...............................................**

**AHSV-7 DQ118704**  **.............................................................G.........................T........................A...............................................**

**AHSV-7 DQ118703**  **.............................................................G.........................T........................A...............................................**

**AHSV-7 AY159954**  **......T......................................................G.........................T........................A...............................................**

**AHSV-7 AY159953**  **.............................................................G.........................T........................A...............................................**

**AHSV-7 AY159952**  **.............................................................G.........................T........................A...............................................**

**AHSV-7 AY159951**  **..............................C..............................G.........................T........................A...............................................**

**AHSV-7 AY159950**  **.............................................................G.........................T........................A...............................................**

**AHSV-7 AY159949**  **.............................................................G.........................T........................A...............................................**

**AHSV-7 AY159948**  **.............................................................G.........................T........................A...............................................**

**AHSV-7 AY159947**  **.............................................................G.........................T........................A...............................................**

**AHSV-7 AY159946**  **.............................................................G.........................T........................A...........C...................................**

**AHSV-7 AY159945**  **....................................................A.......G..........................TA...........T.....................................................T.....**

**AHSV-7 AY159944**  **....A...............................................A.....A.G..........................TA..................................................C..............T.....**

**AHSV-7 AY159943**  **....A...............................................A.....A.G..........................TA..................................................C..............T.....**

**AHSV-7 AY159942**  **....A...............................................A.....A.G..........................TA..................................................C..............T.....**

**AHSV-7 AY159941**  **....A...............................................A.....A.G..........................TA..................................................C..............T.....**

**AHSV-7 AY159940**  **....A...............................................A.....A.G..........................TA..................................................C..............T.....**

**AHSV-7 AY163330**  **................................................................................................................................................................**

**AHSV-1 RSArrah/01** **G.AA.CTT.....T..A.....AC.A.C.G..AGT..GC..T.G..AA.GA.A...G..G.G....G.GCG---..TC.AACC..T.AA........G..GC..A.ACGT..GGA..AAACC.....A..A..AGAT...........A...........**

**AHSV-2 RSArrah/02** **.GGGTTTA....A...A..C..AC.A..AG..AA.A..C.AT..G.A..AA.A..GG.AG.G..TGGGTC.---..C...TCC..T.A...C.....A..T..TA..CGA..AGA...GCC......A..G...GAT.................T.....**

**AHSV-3 RSArrah/03** **..GGT.......GTA.......AGC.G.AG.TAA.A.GTTTT.G..C..G..A...A....G..TG.....AAA..AT.AAAG...TT.........C.CG...A.A..A..CGA..C......C..A.....AG.G........G..............**

**AHSV-4 SPArrah/04** **.GGGT.A.....AA..A....TA...G..G.AA.C.....AT.G...A.A.C....ACCG....TGG.GGA.AA..ATGC.A....T....C.....A..AA......TG....AC..A..T..C.....G....AT......A.A........AACA..**

**AHSV-5 RSArrah/05** **G.AA..AT....AA..TT.C.....A...G.ACAA..GC..T.GG.T..G.C....A....G...A.T..CGAA...TATAA...AAT.G.......G..TA..A..ATG..G.A.AAGA....C..AT.....GA.A.C.....C........T..AA.**

**AHSV-6 RSArrah/06** **.CCA........AG..T....TT..AG.AG..A.T..TC.AA.G..TCG..C...G..AGGC...A.T...AAG..TC.AAAG..T.AA..C.....A..TAG.A.ACT...G....AAA.A............GGT........C........T.....**

**AHSV-8 RSArrah/08** **GCGAA.AT....AAA.AT.....GC.G.AG.CCAA...CGTT.G..T..A.C...GA....G..TGG.GG.GT...TCATT..A...TTG.......A..GA.TA.TATG..AGA.AAAA.A..C..G......CA.A...................GA.**

**AHSV-9 PAKrrah/09** **..GAT..A....AAA.T...GTC..AG.A..CAGA..G.G.A.GC..A.A..A..GAGCGCG..TTCC..CTCA..CT.AAAGA.C.AAG.......C...AG.T.A..T..A....AATTC.....C..G..CGA.......A.A........T.....**

**SRT-8 assay**

**AHSV-8 RSArrah/08** **GGGTGAGCGGATTGTTGATAGCAAAGAGATCGTGTGG-TATGATTTGTCGTTGACCAATTTCGACCTGGTGCGTTCGCAGAATCAATGTTGGATTGGATCAATTTCAAATTTTGAGTTAAGTATGCGATATCACATCATAACCGAAATTTTCCAGAGGTATAGAGTGGATTCAGC**

**AHSV-8 DQ868775**  **........................G............-.........................G........................C......................................................................................**

**AHSV-8 AY163333**  **.....................................-.........................................................................................................................................**

**AHSV-1 RSArrah/01** **A..G..A..AG.GAAA..CGCG..G..AT.G......A.G-...C.T...C..G.G.....T.GGTGTTC...C.GCT.TG...G.......CCA.CT..CTGCGTTG.AGCG...A.CTCCT..A.G..CT.TT.AG.C..ATCT......GCT..A..CCT---TA.CCGT.A**

**AHSV-2 RSArrah/02** **A..C.....TG.GAAG..CGCG......T.A......A.G-.....A..TC.T..........GGT.T.....AAGCT.C...AG...........CGG.CT..GTGG.GGCG...A.TTCGC.CA...T.T.TC.......GTCG..C..TTGT..A...TT---TAC.GGC.A**

**AHSV-3 RSArrah/03** **A.....ATATG.GAAG...GCG..G...C....C...T.C-...A.CG.TAAC..A..C...A..A.CAC....C.TTTTG..AG...C...CC.TCT..TTGCG.GG.GGC.......TCCT..A.G.T...TC.G..T...A.G..C...AC...A...C.---A---GGC.A**

**AHSV-4 SPArrah/04** **A......GAA..AC.....TCG......C....C...A.G-...A....AC.TTTA.....T.GAGC...CA.A..T..CG..AGG..C.....CTCC....GCGTCGCGA.....G.G.A.T.A..TC..GCAC.A...GTTAGG.....TTCAC.C.T.GACA..ATG..G.A**

**AHSV-5 RSArrah/05** **A..C...AAA....G....GCG..G........T...-........A..T.....T.....T.GATGTTCTA.A...TTAG..TCT......G.G..G...G..G.T.GA.C....C.G.A.T....G.TC...C.T...T...C...A..TG.AC.A...CAGCAT...G..CG**

**AHSV-6 RSArrah/06** **A..GC.AAAAG.GAA....TC..TC.CTC.-....AAC..C...C.TG.AC.T..G.......GGG.TTC.A.GCGT...G.CTGT..C.....CTC...GTG.AGTGCCA.....C.TTCG...A..GCCA.......T.T..CT.....TAG...AATCGA--A.ACAAG..A**

**AHSV-7 KENrrah/07** **...A....ATG.GA.....GCA.....AT.A..T...TAT-...A.TG....A..A....AT.GAACAAC...C.ATTTTG....G......CCATCC...TG...C.CCACG..A...TCA...A.....TTTT.G..T..T.....C..T.....A.....---.AC.GAC.A**

**AHSV-9 PAKrrah/09** **C..A...AAAG.GAAG..CTCGC...C.C........-......C.TG.T.....T....A.ACA..A.....GC.A..AG..AG.........AATG..GTG.A.TG...C...A.AC.C.T..A...TCGCG..G..C..TATG..A...G..C.C.TATC--..AGGAG.CT**

**SRT-9 assay**

**AHSV-9 PAKrrah/09** **TTGTATCATATTGGTATCGAGTTCGACAGGTTCAAACCTCAAAGG---GTATTGAGAGGAGG---TCGATTGAGGATGTCAAA---TATAGGGAGTTTGATATAGAAAGTTTTAAGCCTTACGCTATTGGCGAGATCGGTATTCACGCATCAACTTACA**

**AHSV-9 DQ868776**  **..................................G..........---..GC.........A---..................---......................................G.....T............................**

**AHSV-9 AF043926**  **.............................................---..............---..................---..............................................................G..........**

**AHSV-1 RSArrah/01** **....T..T.......TCA..A.AGA...TT..TCG.TTA.G.CA.CA-AA.AA.C..ACT.A--CATTCGA..TA.AAAG.CTGAA....AC........CT.C...GAC..C.....GGCATG......A..AT.G..G..A.....G..G..A..T.**

**AHSV-2 RSArrah/02** **.......T.......TCA..A..GA..GCACAACGCA.GT.....GA-.GGGGA....T..A--TATTCGG..TCGAAAA.C.GGA..CCA.C....C...G.....GA......A...GCAAG.G.A..G..AC.T..AT.......G.....A..T.**

**AHSV-3 RSArrah/03** **....C............GA.T...A.A.A...ACT.TTGAGGCT.AT-TCGAA.C..TTG.A--CG.ACG..GC..ACAT.T.CAG...CAT...A.A...G.T...GA...C..A....G...G....CG..AC.T..AT.A..TTGC.....C..T.**

**AHSV-4 SPArrah/04** **.C...............A..A.ATC...A...G..GTAA.G....CGC...A...AGTTCT.GACAT..A....A.ACAG..GCCG...TTT..A.....ATAT..TGA...C..A..C.GTT.A.....A...T.G..G..C..T.....C..A..T.**

**AHSV-5 RSArrah/05** **.CA.T..T.........AA.A.AACTA.....G..G.AAGG..TCTATTG.C.TTA.CTGAT---ATAGGG.G.....CA...AAG....CAC........CCT..TGA......A..AATG...G.G.CT...C.G..AGCA.....G..G..G..TG**

**AHSV-6 RSArrah/06** **....T..G...........T...GAGA.AA.---...AA.G...C---ACC.....TTTTTA---A.TGAA...A..CGA...---GTCGC.C........T.T..GGA..A...A..GATG........A.....G..G.....T..G..G..A..T.**

**AHSV-7 KENrrah/07** **....T..G.........ACGT..GAGA....GG.GGTTGGTCG..CG-A.GGC....ATC.A--TATTCA...TCGA.AG.CCAAG.T.TCC.....C...G.C..TGAC.A...A..A.GTT.AG.G.CG..A..G..GC.A..TT..........T.**

**AHSV-8 RSArrah/08** **....TA.C.........AA.A..AC.AG...GG.G.AGAA.G.TTTACTC....T..ATGAT---ATCTAC..T..AAAA.CGGAA...CA.C........CCG..TGA...C.....AATG.T......A.....G...G.......T.....A..T.**

**Figure S1: Nucleotide alignments for the design of AHSV virus-species (Seg-1 and Seg-3) and type-specific (Seg-2) assays.**

Seg-1 and Seg-3 indicate the virus-species-specific assays while SRT-1 to SRT-9 indicate respective type-specific assays. The nucleotide sequence of primers and probes for individual assays, including redundant bases, are listed in Table 2.

**
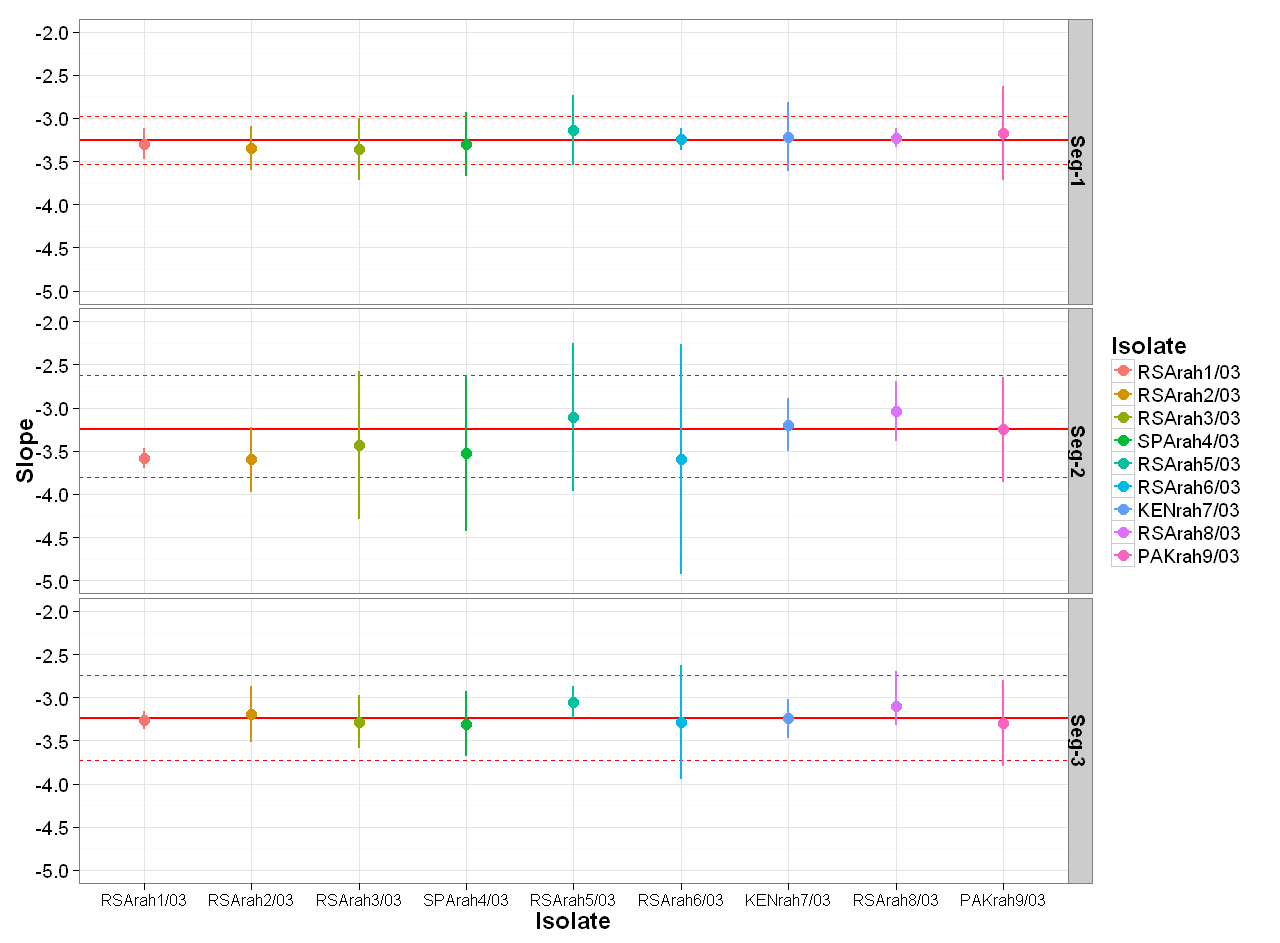
**

**Figure S2: Slopes values in relation to the slope of the common regression line for virus-species and type-specific RT-PCR assays.**

Comparison of slopes of the nine reference strains to the respective common regression line in virus-species-specific (Seg-1 and Seg-3) and type-specific (Seg-2) assays with corresponding 95% CI. Slopes of all nine serotypes of AHSV are highly similar in each of the virus-species-specific or individual type-specific assays respectively. Solid red line represents common regression line while dotted lines mark defines the 95% CI.
